# Ceftazidime–Avibactam Versus Colistin-Based Regimens for Carbapenem-Resistant Enterobacterales Bloodstream Infections: A Six-Year Retrospective Cohort and 30-Day Mortality Analysis

**DOI:** 10.3390/microorganisms14092102

**Published:** 2026-09-19

**Authors:** Victor-Pierre Ormeneanu, Anca Zanfirescu, Corina Andrei, Florin Dumitru, Flavius Anghel, Răzvan Adam, Simona Negreș

**Affiliations:** 1 Department of Pharmacology and Clinical Pharmacy, Faculty of Pharmacy, “Carol Davila” University of Medicine and Pharmacy, Traian Vuia 6, 020956 Bucharest, Romania; victor-pierre.ormeneanu@rez.umfcd.ro (V.-P.O.); corina.andrei@umfcd.ro (C.A.); simona.negres@umfcd.ro (S.N.); 2 Elias Emergency University Hospital, 011461 Bucharest, Romania; dumitruflorin1978@gmail.com (F.D.); flavius.anghel27@gmail.com (F.A.); razvan.adam@prof.utm.ro (R.A.)

**Keywords:** bloodstream infection, carbapenemase-producing Enterobacterales, ceftazidime–avibactam, colistin, metallo-β-lactamase, *Klebsiella pneumoniae*, OXA-48, NDM, propensity score

## Abstract

Bloodstream infections (BSIs) caused by carbapenemase-producing Enterobacterales (CPE) carry a high mortality, and comparative real-world data remain limited, particularly for metallo-β-lactamase (MBL) and dual-carbapenemase producers. We conducted a single-centre, six-year (2020–2025) retrospective study of 308 adults with CPE BSIs at a Romanian tertiary hospital, applying multivariable logistic regression, Cox proportional-hazards models with a 72 h landmark analysis, and inverse-probability-of-treatment-weighted (IPTW) regression to examine 30-day mortality and microbiological recurrence. Pre-specified sensitivity analyses addressed calendar period, carbapenemase class and incomplete outcome ascertainment. *Klebsiella pneumoniae* accounted for 92.5% of isolates, MBL determinants for 44.1% and dual carbapenemases for 31.8%. Thirty-day mortality was 72% among 275 patients with documented day-30 status. Vasopressor requirement, viral pneumonia and bacterial respiratory infection were independently associated with death, whereas definitive ceftazidime/avibactam with or without aztreonam (C/AVI ± AZT) was associated with lower mortality (adjusted OR 0.38; 95% CI 0.17–0.83). Mortality was 66.3% with C/AVI ± AZT, 79.3% with colistin and 67.1% with other regimens. After IPTW, C/AVI ± AZT was associated with lower 30-day mortality than colistin (OR 0.48; 95% CI 0.26–0.93); in the OXA-48/KPC subgroup, the estimate was unchanged but less precise (OR 0.49; 95% CI 0.20–1.19). The association was attenuated and no longer significant after adjustment for calendar period (OR 0.72; 95% CI 0.37–1.42), with epoch-stratified estimates of 1.14, 0.75 and 0.44 for 2020–2021, 2022–2023 and 2024–2025, respectively; crude mortality under colistin was stable across epochs (81.1%, 76.6%, and 81.2%, respectively), whereas mortality under C/AVI ± AZT fell (83.3%, 72.2%, and 60.7%, respectively). Weighted estimates were near-identical in isolates with and without an MBL determinant (0.46 and 0.48, respectively). Recurrence (10.1%) was more frequent with single OXA-48 or MBL producers. C/AVI ± AZT was associated with lower 30-day mortality than colistin, but the association was concentrated in the period when the agent was routinely available and did not survive adjustment for calendar time. These findings describe an association, not a causal survival advantage.

## 1. Introduction

Antimicrobial resistance among Enterobacterales is one of the leading global health threats. Carbapenem-resistant Enterobacterales are classified as critical-priority pathogens by the World Health Organization, with carbapenem-resistant *Klebsiella pneumoniae* ranked among the highest-priority pathogens in the 2024 Bacterial Priority Pathogens List [1]. Following the COVID-19 pandemic, the epidemiology of these organisms has shifted, with metallo-β-lactamase (MBL) and dual-carbapenemase producers increasingly reported alongside, and in some settings replacing, KPC producers [2].

Carbapenem resistance in Enterobacterales is chiefly mediated by three clinically important carbapenemase groups: KPC, OXA-48-like enzymes, and MBLs (predominantly NDM). Hospitals in the Middle East and North Africa, as well as in Eastern Europe, have increasingly reported dual OXA-48 + NDM *Klebsiella pneumoniae* [3,4]. Bloodstream infection is among the most severe presentations of infection with these organisms, with 30-day mortality frequently exceeding 40–50% [5].

β-lactam/β-lactamase-inhibitor combinations, principally ceftazidime/avibactam (C/AVI), are now guideline-endorsed treatment options for KPC- and OXA-48-producing carbapenem-resistant Enterobacterales [6], and observational data support improved survival with these agents [7]. For MBL producers, which are not inhibited by avibactam alone, the combination of aztreonam with avibactam (or ceftazidime/avibactam plus aztreonam) has emerged as a preferred treatment option [8], while cefiderocol represents an alternative in selected cases [9]. Colistin, long used as a last-resort agent, is limited by unpredictable pharmacokinetics, nephrotoxicity, and uncertain efficacy [10,11,12].

Despite these advances, real-world comparative data remain scarce, particularly from high-burden endemic settings and for MBL and dual-carbapenemase producers. The comparative effectiveness of C/AVI ± AZT versus colistin across carbapenemase types remains incompletely defined. We therefore conducted a six-year single-centre study describing the characteristics and outcomes of adults with CPE bloodstream infection and comparing definitive therapy with C/AVI ± AZT against colistin, with a pre-specified sensitivity analysis restricted to OXA-48- or KPC-producing isolates. Further pre-specified sensitivity analyses addressed calendar period, carbapenemase class and incomplete ascertainment of day-30 vital status.

## 2. Materials and Methods

### 2.1. Study Design and Population

A six-year retrospective observational study was conducted between 1 January 2020 and 31 December 2025 at Elias Emergency University Hospital, Bucharest, Romania. Patients were eligible if they were aged ≥ 18 years, had at least one positive blood culture documented in the microbiological reports, had received antimicrobial treatment for the infection, and had an identified resistance mechanism. We excluded blood cultures judged to represent contamination or transient or asymptomatic bacteraemia and those from patients who had died within 24 h of blood culture collection. Demographic, clinical, epidemiological, microbiological, and treatment characteristics, as well as laboratory findings on the day of blood culture collection, were retrieved from the electronic medical records. Vital status at day 30 was ascertained from the hospital electronic record and, for patients discharged or transferred before day 30, from the electronic hospital database. Patients for whom day-30 status could not be established by either source were retained in the descriptive analysis but excluded from mortality models; these patients were compared with those with documented day-30 status as described in Section 2.5.

Because the study period spanned the COVID-19 pandemic and a period of changing antimicrobial availability, calendar period was defined a priori as three approximately equal epochs: 2020–2021 (*n* = 110), 2022–2023 (*n* = 93) and 2024–2025 (*n* = 105). Access to ceftazidime/avibactam broadened progressively across these epochs, whereas aztreonam availability remained intermittent throughout the study period. Availability of these antibiotics was assessed using hospital pharmacy records and antibiotic-consumption reports documenting procurement history. Calendar period was included both as a covariate and as a stratification variable in the analyses described in Section 2.5.

### 2.2. Microbiological Methods

Blood cultures were processed according to the routine standard operating procedures of the hospital microbiology laboratory, which applies European Committee on Antimicrobial Susceptibility Testing (EUCASTBreakpoint Tables, version 14.0) clinical breakpoints and guidance documents, updated annually to the version current at the time of testing. Bacterial identification was performed using an automated system (VITEK, bioMérieux, Marcy-l’Étoile, France) and subsequently confirmed by matrix-assisted laser desorption/ionisation time-of-flight mass spectrometry (MALDI-TOF MS, Bruker Daltonics, Bremen, Germany). Extended antimicrobial susceptibility testing was performed by disc diffusion, complemented where appropriate by automated broth-based systems, and results were confirmed by broth microdilution when equivocal or when a reference method was required for reliable interpretation. Colistin susceptibility was determined by broth microdilution in accordance with EUCAST recommendations. Carbapenemase production was confirmed in all included isolates, and the enzyme class was established by rapid immunoassay-based carbapenemase detection (NG-TEST^®^ CARBA-5, (NG Biotech, Guipry-Messac, France) and multiplex PCR (BioFire panel, bioMérieux). Isolates were classified as single producers (KPC, OXA-48-like or metallo-β-lactamase) or as dual producers (OXA-48-like + NDM, KPC + OXA-48-like, KPC + NDM) based on these results. Multilocus sequence typing and whole-genome sequencing were not performed. Ceftazidime/avibactam was not included in the routine extended susceptibility panel throughout the study period. However, a phenotypic susceptibility result was retrievable from the laboratory records for 300 of the 308 isolates, including every isolate from a patient treated with ceftazidime/avibactam; these data are reported in Section 3.7.The eight isolates without an available phenotypic result all carried an MBL determinant. Isolates were classified for the primary analyses according to confirmed carbapenemase class, while the correspondence between carbapenemase class and phenotype was examined separately.

### 2.3. Antimicrobial Therapy and Dosing

Antimicrobial therapy was prescribed by the treating physicians in consultation with the hospital infectious-diseases service, in accordance with institutional protocols aligned with the Summary of Product Characteristics and international guidance. Initial (empirical) therapy was defined as the regimen administered between collection of the index blood culture and availability of the carbapenemase result, whereas definitive therapy was defined as the regimen administered for at least 48 h thereafter. Definitive ceftazidime/avibactam was given as monotherapy or in combaination with aztreonam, colistin, or both; aztreonam was added only for isolates carrying a MBL determinant. Because carbapenemase class was established by rapid immunoassay and multiplex PCR performed on the positive blood culture, the resistance determinant was identified within hours of culture positivity. Consequently, aztreonam was prescribed as part of the initial definitive regimen rather than added later as an escalation. No patient received aztreonam empirically before the carbapenemase result was available, and all patients who received it had an MBL determinant. The decision to administer aztreonam was based on the carbapenemase result alone: aztreonam–avibactam synergy testing was neither available nor performed, and aztreonam minimum inhibitory concentrations were not used to guide treatment. Renal dose adjustment of colistimethate sodium followed international consensus guidelines for the optimal use of polymyxins [13]. Ceftazidime/avibactam administered alone was dosed at 2.5 g three times daily by extended infusion over two to three hours, with dose reduction according to creatinine clearance as specified in the Summary of Product Characteristics and international guidance. Aztreonam was likewise adjusted for renal function. When administered in combination, ceftazidime/avibactam and aztreonam were infused simultaneously through separate lines over three hours at standard maintenance doses of 2.5 g and 2 g three times daily, respectively. Colistin was initiated with a 9 MIU loading dose followed by 4.5 MIU twice daily. Therapeutic drug monitoring of colistin was not available at our institution, and plasma concentrations were therefore not measured. Because dose, infusion duration, and renal dose adjustments were recorded in free-text prescription notes rather than a structured field, these variables could not be abstracted reliably for every patient and are therefore not reported as distributions. The composition and duration of definitive therapy and the timing of ceftazidime/avibactam initiation were retrievable and are reported in Section 3.3 and Section 3.7.

### 2.4. Definitions

Persistent or recurrent bacteraemia was defined as isolation, within 28 days after the index culture, of an identical organism harbouring the same carbapenemase gene and exhibiting the same resistance pattern. Because neither multilocus sequence typing nor whole-genome sequencing was available, this operational definition cannot distinguish microbiological persistence of the index strain from reinfection with a genetically distinct isolate sharing the same species, carbapenemase gene, and antibiogram; these events were therefore analysed as a single composite endpoint. The source of BSI was defined as the anatomical site or device from which an organism microbiologically identical to the blood isolate was recovered at or before the index date. Primary bacteraemia was defined as BSI with no identifiable source. Seven-day mortality was defined as death within seven days of the index bloodculture collection. Inadequate therapy was defined as treatment that included an antimicrobial to which resistance was documented by susceptibility testing or whose use was discouraged by international guidelines. Immunosuppression was defined as more than three days of in-hospital corticosteroid therapy, chronic corticosteroid or biological therapy, recent chemotherapy, or neutropenia.

### 2.5. Statistical Analysis

Continuous variables are reported as median and interquartile range (IQR) and categorical variables as counts and percentages. Associations between categorical variables were tested using the χ^2^ test or Fisher’s exact test, as appropriate. Continuous variables were compared using Student’s *t* test (for normally distributed variables) and the Mann–Whitney U test (for non-normally distributed variables). Univariate analyses of factors associated with mortality at each time point included only patients with documented survival status. For secondary outcomes, binomial logistic regression models included predictors identified in univariate analysis together with clinically relevant or literature-supported variables, irrespective of statistical significance in univariate analyses.

Because the interval between the index blood culture collection and initiation of the first active antimicrobial could not be reconstructed precisely, one Cox proportional-hazards model was fitted using initial therapy as the treatment exposure. To address immortal-time bias in the analysis of definitive therapy, and to examine the association between subsequent treatment with C/AVI ± AZT and outcome, a 72 h landmark analysis was performed. Patients who died or were discharged before 72 h were excluded from this analysis, and a second Cox model was fitted from that point onwards. The proportional-hazards assumption was assessed using graphical inspection of log-rank curves for categorical variables and the time-dependent covariate method for continuous variables. Age was the only variable that did not satisfy the proportional-hazards assumption and was therefore retained in the Cox models with an age × ln(time) term.

To explore the association between definitive treatment and the primary outcome, a propensity score (PS) was estimated in the subgroup of 202 patients eligible for the weighted comparison. The PS model included variables considered clinically relevant or likely to contribute to confounding by indication: age and Charlson comorbidity index (CCI) as indicators of chronic-disease burden; carbapenemase class and source of bloodstream infection; indicators of acute disease severity, namely, vasopressor requirement and intensive care unit (ICU) stay or admission, used in the absence of dedicated scores such as SOFA; and calendar period (2020–2021, 2022–2023, and 2024–2025), included to account for the changes in ceftazidime/avibactam availability and pandemic-related differences in case mix and management. Stabilised inverse-probability-of-treatment weighting(IPTW) was used because of the limited sample size. The adequacy of covariate balance was assessed using standardised mean difference (SMD) before and after weighting; an absolute SMD < 0.1 was considered indicative of acceptable balance (Table 1). A sensitivity analysis using the same methodological framework was performed in the subgroup of 110 patients infected with OXA-48- or KPC-producing isolates, for which ceftazidime/avibactam would generally be expected to be active in the absence of additional resistance mechanisms.

Three additional pre-specified sensitivity analyses were performed. First, calendar period was included in both the PS model and the weighted outcome model, and the weighted analysis was repeated separately within each calendar epoch. Second, the weighted analysis was repeated within strata defined by carbapenemase class (OXA-48-like and/or KPC without an MBL determinant vs. isolates carrying an MBL determinant). Definitive therapy was also modelled as a five-level categorical exposure (colistin-based therapy, ceftazidime/avibactam alone, ceftazidime/avibactam with colistin, ceftazidime/avibactam with aztreonam, and ceftazidime/avibactam plus both colistin and aztreonam) in a multivariable logistic model adjusted for age, CCI, carbapenemase class, vasopressor requirement, ICU stay and source of infection. Firth penalised estimates were reported alongside conventional estimates because of the small size of some regimen groups. The interval between index blood culture collection and the initiation of ceftazidime/avibactam was analysed within the treated arm both categorically and continuously. Polymicrobial bloodstream infection was then added to the PS model, and the weighted analysis was repeated separately for monomicrobial and polymicrobial episodes. Third, patients with and without documented day-30 status were compared with respect to all covariates included in the PS model, definitive treatment allocation, and calendar period. The primary weighted estimate was recomputed under two extreme assumptions, with all patients of unknown status assigned first to survival and then to death.

Throughout, treatmenteffect estimates are reported and interpreted as associations. Treatment was allocated by the responsible clinicians rather than by randomisation; validated severity-of-illness scores such as SOFA or APACHE II were not recorded and were replaced by the pre-specified clinical surrogates described above; and the interval between index blood culture collection and initiation of the first active antimicrobial could not be reconstructed. The models therefore account only for measured confounding and do not support causal inference.

All analyses were performed with IBM SPSS Statistics for Windows, version 23.0. A two-sided *p* value < 0.05 was considered statistically significant. Thisstudy is reported in accordance with the Strengthening the Reporting of Observational Studies in Epidemiology (STROBE) statement.

## 3. Results

### 3.1. Demographic Characteristics and Clinical Features at Admission

**Figure 1:**
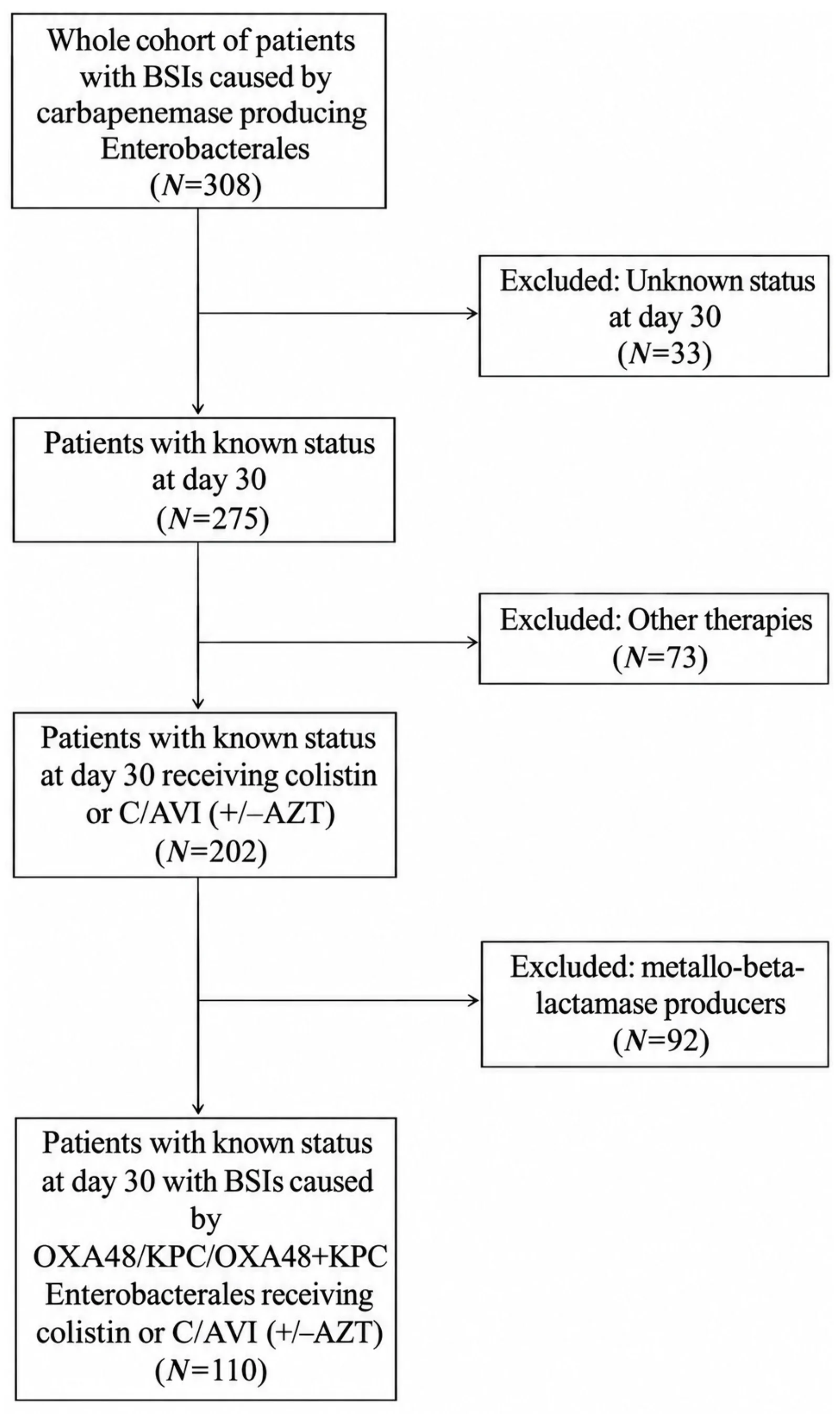
Flow diagram of the study population (STROBE). AZT aztreonam, BSI bloodstream infection, C/AVI ceftazidime/avibactam.

In total, 308 patients were included in the main analysis, of whom 56.8% were male (Figure 1). Median age and CCI in the cohort were 69 years (IQR 58–77) and 6 (IQR 4–8), respectively. Median in-hospital and ICU length of stay were 27 days (IQR 15–44) and 12 days (IQR 4–25), respectively. The median time from admission to index blood culture was 14 days (IQR 8–25). At the time of blood culture collection, 71.1% of patients were admitted to an ICU and 36.7% had undergone a recent surgical intervention. Prior to bloodstream infection, 98.7% of patients had a history of antibiotic exposure within the previous three months (Table 2).

### 3.2. Microbiological Characteristics

Carbapenemases were distributed as follows: double producers (OXA-48-like + NDM 27.92%, KPC + OXA-48 2.59%, and KPC + NDM 1.29%) and single producers (KPC 30.19%, MBL 14.93%, and OXA-48 23.05%). *Klebsiella pneumoniae* was the major causative agent (92.53%) and was also the only pathogen harbouring multiple carbapenemase genes. Besides *Klebsiella* species, the MBL-producer category included NDM-carrying *Proteus mirabilis*, *Serratia marcescens*, *Providencia stuartii*, *Escherichia coli* and *Enterobacter cloacae* and one VIM-producing *Citrobacter freundii*.

### 3.3. Antimicrobial Treatments

Overall, 76% of patients received an inappropriate initial therapy. Empirical regimens comprised antipseudomonal β-lactams (piperacillin/tazobactam or carbapenems) in 43.5% of patients, colistin in 31.8%, ceftazidime/avibactam in 10.38%, and other antibiotics (aminoglycosides, fluoroquinolones, and tigecycline) in 14.32%. Definitive regimens comprised C/AVI ± AZT in 31.8% of patients, colistin in 41.9%, antipseudomonal β-lactams in 18.18% and other therapies in 8.12% (Table 2).Of the 98 patients who received definitive C/AVI ± AZT, 29 received ceftazidime/avibactam alone, 15 received ceftazidime/avibactam in association with aztreonam, 35 received ceftazidime/avibactam plus colistin and 19 received ceftazidime/avibactam plus both aztreonam and colistin; every patient who received aztreonam carried an MBL determinant, and, in all cases, aztreonam was initiated concomitantly with ceftazidime/avibactam once the rapid carbapenemase assay had identified the determinant; aztreonam was never added subsequently as an escalation. The interval from index blood culture collection to initiation of the ceftazidime/avibactam-based regimen was a median of 2 days (IQR 1–3) in patients who received aztreonam and 2 days (IQR 0–4) in those who did not (*p* = 0.99), indicating no difference in the timing of initiation between the two groups. Of the 50 patients whose isolates carried an MBL determinant, 34 received aztreonam and 16 did not. The median duration of definitive therapy was 10 days (IQR 6–13).

### 3.4. Primary and Secondary Outcomes

Among the 308 patients in the cohort, day-30 vital status was documented for275. Overall 30-day mortality among patients with documented status was 72%, corresponding to 64.3% when calculated against the entire cohort. The remaining 33 patients (10.7%) had unknown day-30 vital status and were therefore excluded from mortality analyses.

By definitive regimen, 30-day mortality was 66.3% (57/86) among patients receiving C/AVI ± AZT, 79.3% (92/116) among those receiving colistin, and 67.1% (49/73) among those receiving other therapies. Among patients with documented day-30 status who had MBL-producing isolates, 30-day mortality was 70.6% (89/126); these patients accounted for 44.9% of all deaths among patients with known day-30 status.

In the multivariable analysis of 275 patients, vasopressor requirement (aOR 7.39; 95% CI 3.43–15.91), viral pneumonia (aOR 3.56; 95% CI 1.32–9.63), and bacterial respiratory infection (aOR 2.12; 95% CI 1.03–4.38) were independently associated with increased mortality, whereas definitive treatment with C/AVI ± AZT was associated with lower mortality (aOR 0.38; 95% CI 0.17–0.83) (Table 3). After calendar period was added to the model, the association between C/AVI ± AZT and lower mortality was attenuated and no longer reached conventional statistical significance (aOR 0.44; 95% CI 0.19–1.02; *p* = 0.055), while the estimates for vasopressor requirement, viral pneumonia and bacterial respiratory infection remained essentially unchanged. Univariate analyses of 30-, 7- and 14-day mortality and the corresponding multivariable models are presented in Tables S1–S5.

Mortality was 34.3% (104/303) at day 7 and 54.9% (163/297) at day 14. Vasopressor requirement was the strongest independent predictor of mortality at both time points (aOR 6.41; 95% CI 3.30–12.44 at day 7 and aOR 6.37; 95% CI 3.33–11.98 at day 14). Recent chemotherapy was independently associated with 7-day mortality (aOR 4.10; 95% CI 1.30–12.93). Definitive treatment with C/AVI ± AZT remained associated with lower mortality than colistin at both time points (aOR 0.44; 95% CI 0.22–0.89 at day 7 and aOR 0.36; 95% CI 0.18–0.73 at day 14) (Tables S2, S3 and S5).

Bacterial persistence or recurrence was observed in 10.1% of cases and occurred after a median of 4 days (IQR 2–6). On univariate analysis, no variable was significantly associated with recurrence, although recurrence was more frequent among patients receivingcorticosteroids at the onset of infection (13.9% vs. 7.5%; *p* = 0.08) (Table 4. In the multivariable model, a shorter interval from hospital admission to the index bloodstream infection was independently associated with recurrence (aOR 0.94 per day; 95% CI 0.90–0.98). Recovery of the pathogen in follow-up cultures was also more frequent among patients infected with single MBL- or OXA-48-producing isolates than among those infected with KPC-producing isolates or co-carriers (KPC/OXA-48 + NDM) (aOR 2.46; 95% CI 1.12–5.41) (Table 5).

### 3.5. Survival Analysis and Cox Proportional-Hazards Regression

**Figure 2:**
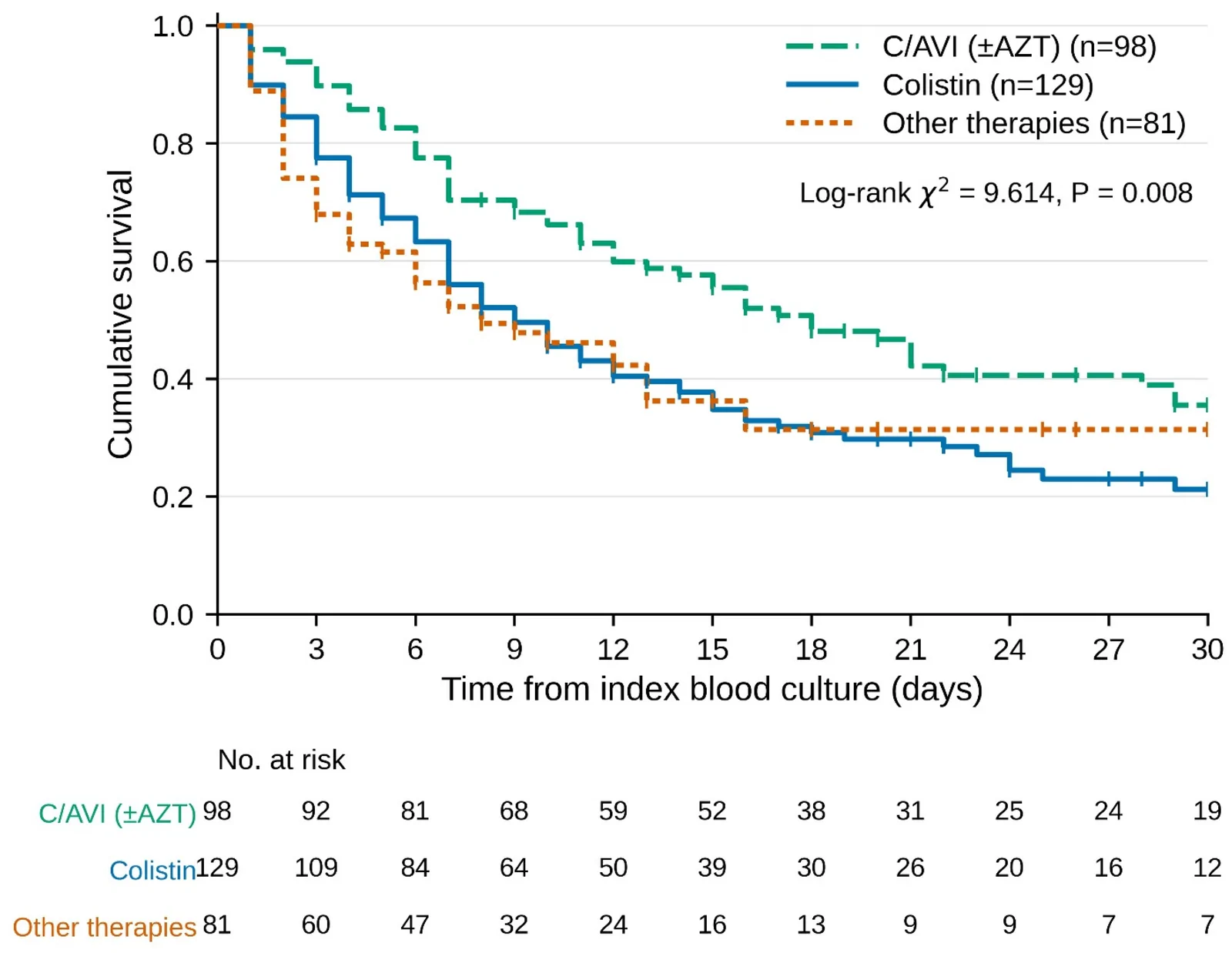
Kaplan–Meier estimates of 30-day survival by definitive treatment in the whole cohort (*n* = 308, C/AVI ± AZT *n* = 98, colistin *n* = 129, other therapies *n* = 81; log-rank χ^2^ = 9.614; *p* = 0.008). AZT aztreonam, C/AVI ceftazidime/avibactam.

**Figure 3:**
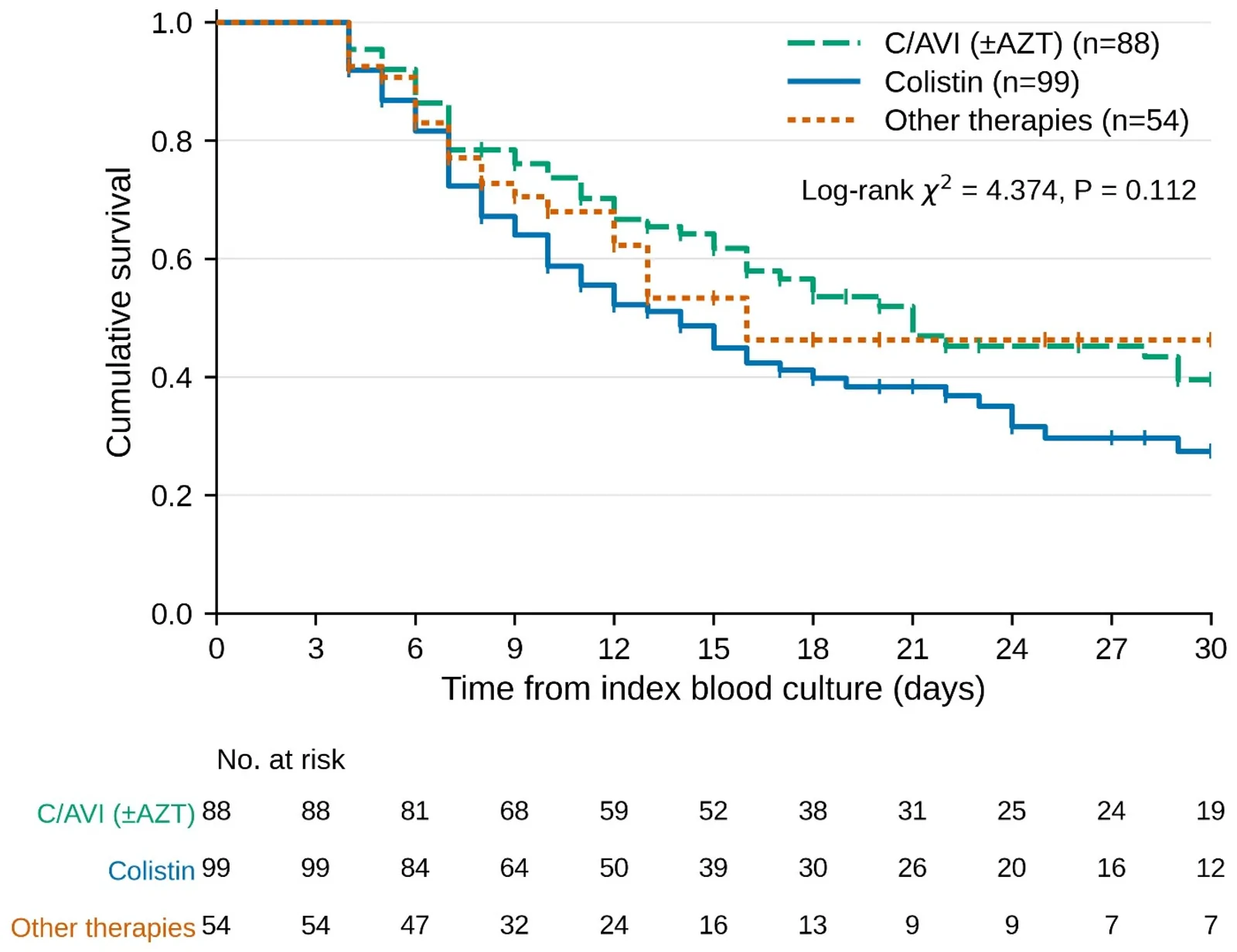
Kaplan–Meier estimates of 30-day survival by definitive treatment in the 72 h landmark analysis (*n* = 241, C/AVI ± AZT *n* = 88, colistin *n* = 99, other therapies *n* = 54; log-rank χ^2^ = 4.374; *p* = 0.112). AZT aztreonam, C/AVI ceftazidime/avibactam.

Crude survival analysis using the log-rank test in the whole cohort of 308 patients identified significant differences in survival distributions across definitive treatment groups (Figure 2; χ^2^ = 9.614; *p* = 0.008). Conversely, in the 72 h landmark analysis, which was restricted to the 241 patients remaining at risk at that time point, no significant difference was found (Figure 3; χ^2^ = 4.374; *p* = 0.112). In the Cox regression analysis of the whole cohort, older age [retained with an age × ln(time) interaction term] and vasopressor requirement (aHR 1.54; 95% CI 1.12–2.12) were associated with shorter survival; no variable was significantly associated with longer survival, although a urinary source showed a borderline association with longer survival (aHR 0.60; 95% CI 0.36–1.09) (Table 6). In the 72 h landmark model, the direction of association between vasopressor requirement and survival was preserved, although it no longer reached statistical significance (aHR 1.28; 95% CI 0.86–1.89). Other sources of BSI (including biliary, cutaneous, and soft tissue sources) showed a borderline association with shorter survival (aHR 2.18; 95% CI 0.99–6.88) (Table 7).

### 3.6. Outcomes in Patients Receiving Ceftazidime/Avibactam or Colistin

**Figure 4:**
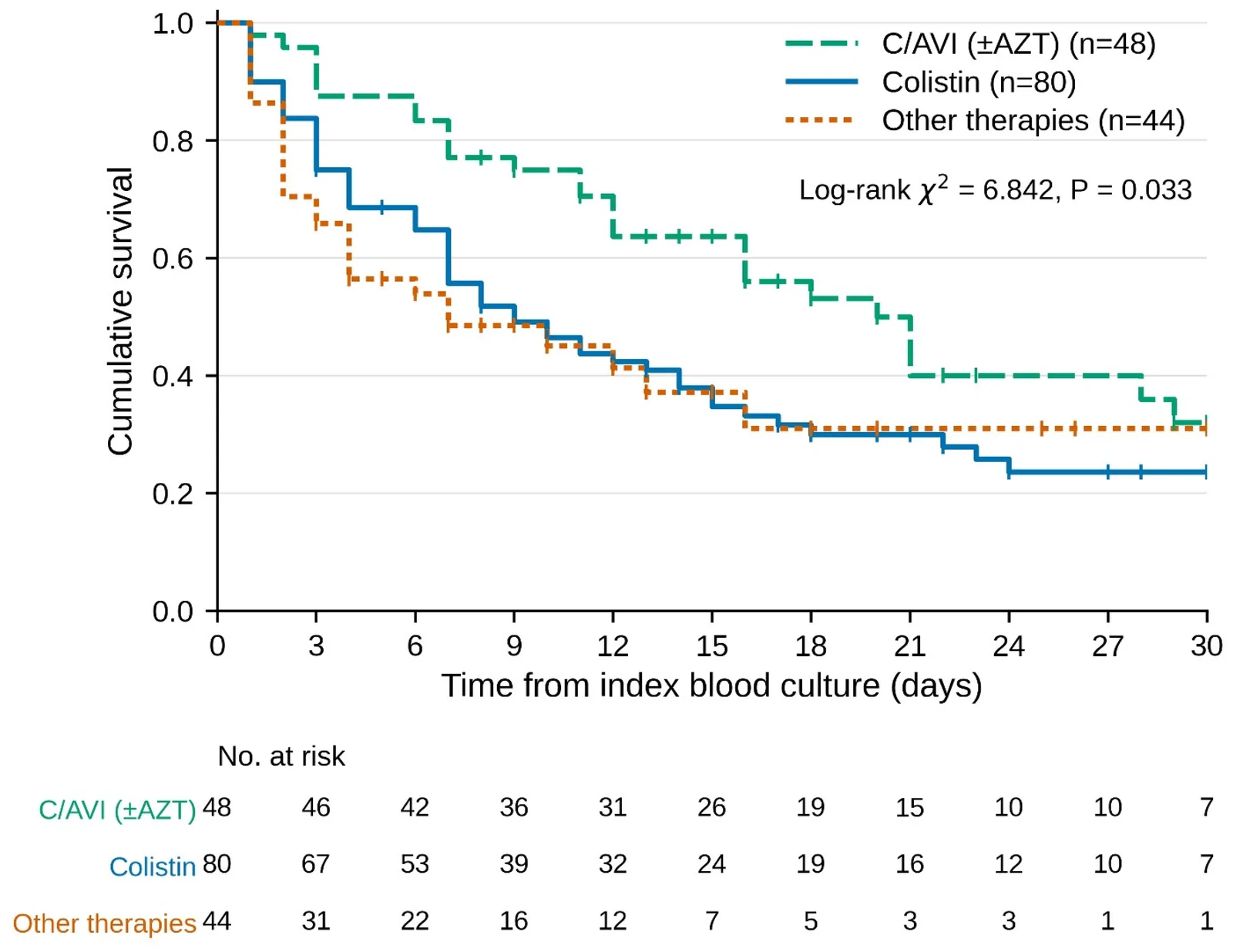
Kaplan–Meier estimates of 30-day survival by definitive treatment in patients infected with OXA-48- or KPC-producing isolates without a metallo-β-lactamase determinant (*n* = 172, C/AVI ± AZT *n* = 48, colistin *n* = 80, other therapies *n* = 44; log-rank χ^2^ = 6.842; *p* = 0.033). AZT aztreonam, C/AVI ceftazidime/avibactam.

In the IPTW-weighted logistic regression, definitive therapy based on ceftazidime/avibactam was associated with lower 30-day mortality than definitive therapy based on colistin (OR 0.48; 95% CI 0.26–0.93) (Table 8). In the sensitivity analysis restricted to OXA-48- or KPC-producing isolates, the point estimate was essentially unchanged, while the confidence interval widened and included unity (OR 0.49; 95% CI 0.20–1.19) (Table 9). Among all 172 patients infected with OXA-48- or KPC-producing isolates without an MBL determinant, including those treated with other regimens, descriptive Kaplan–Meier analysis showed a significant difference in survival across definitive-treatment strata, consistent with the pattern observed in the whole cohort (Figure 4; χ^2^ = 6.842; *p* = 0.033).

### 3.7. Sensitivity Analyses: Calendar Period, Carbapenemase Class and Incomplete Outcome Ascertainment

Adding calendar period to the PS and outcome models attenuated the association between definitive C/AVI ± AZT and 30-day mortality, which no longer reached significance (OR 0.72; 95% CI 0.37–1.42; *p* = 0.347), compared with the primary weighted estimate of 0.48 (95% CI 0.26–0.93; *p* = 0.028). Epoch-stratified weighted estimates were 1.14 (95% CI 0.17–7.69) for 2020–2021, 0.75 (95% CI 0.20–2.73) for 2022–2023 and 0.44 (95% CI 0.16–1.21) for 2024–2025. All estimates were imprecise, particularly that for 2020–2021, which was based on only 12 patients treated with C/AVI ± AZT (Table S8).

These findings coincided with substantial changes in treatment patterns and local epidemiology across the study period. The proportion of patients receiving definitive C/AVI ± AZT increased from 13.6% in 2020–2021 to 22.6% in 2022–2023 and 59.0% in 2024–2025, while the proportion of isolates carrying an MBL determinant increased from 15.5% to 49.5% and 69.5%, respectively, and the proportion of KPC producers decreased from 56.4% to 6.7% (Table S6). Crude 30-day mortality among patients receiving colistin remained stable across the three epochs (43/53, 81.1%; 36/47, 76.6%; and 13/16, 81.2%), whereas mortality among those receiving C/AVI ± AZT decreased from 10/12 (83.3%) to 13/18 (72.2%) and 34/56 (60.7%). Overall 30-day mortality among patients with documented status decreased from 77.9% to 70.2% and 67.7%.

Weighted estimates were similar across the two carbapenemase strata. Among the 110 patients infected with OXA-48-like or KPC-producing isolates without an MBL determinant, the OR was 0.48 (95% CI 0.20–1.15), with crude 30-day mortality of 67.5% (27/40) among those receiving C/AVI ± AZT versus 80.0% (56/70) among those receiving colistin. Among the 92 patients whose isolates carried an MBL determinant, the corresponding OR was 0.46 (95% CI 0.18–1.20), with crude mortality of 65.2% (30/46) versus 78.3% (36/46), respectively (Table S9). The treatment association was therefore similar in the two carbapenemase strata, although both estimates were imprecise.

Because the composition of definitive therapy was available for all patients, treatment was also modelled as a five-level categorical exposure, with colistin-based therapy as the reference category (Table S7). Thirty-day mortality was 79.3% (92/116) with colistin, 60.9% (14/23) with ceftazidime/avibactam alone, 78.8% (26/33) with ceftazidime/avibactam plus colistin, 41.7% (5/12) with ceftazidime/avibactam plus aztreonam, and 66.7% (12/18) with ceftazidime/avibactam plus both aztreonam and colistin. After adjustment for age, CCI, carbapenemase class, vasopressor requirement, ICU stay, and source of infection, the odds of death relative to colistin were 0.27 (95% CI 0.09–0.82) for ceftazidime/avibactam alone, 0.09 (95% CI 0.02–0.43) for ceftazidime/avibactam plus aztreonam, and 0.50 (95% CI 0.12–2.05) for ceftazidime/avibactam plus both agents. Firth penalised estimates were 0.29, 0.93, 0.11, and 0.52, respectively. The lower odds of death were therefore observed in the colistin-free regimens. The aztreonam-containing estimate was the lowest but was based on only 12 patients with documented day-30 status and was consequently imprecise.

Among the 83 treated patients for whom both treatment timing and outcome were documented, theinterval from index blood culture collection to initiation of ceftazidime/avibactam was a median of 2 days (IQR 0–3) and was not associated with 30-day mortality. Crude mortality was 61.1% among patients starting treatment within 0–1 day, 69.2% among those starting on day 2, and 73.5% among those starting after day 2 (*p* = 0.54); the adjusted odds ratio per additional day was 1.12 (95% CI 0.85–1.46).

Phenotypic ceftazidime/avibactam susceptibility was available for 300 of the 308 isolates and corresponded closely to enzyme class: 171 of 172 isolates (99.4%) producing KPC or OXA-48-like enzymes without an MBL determinant were susceptible, compared with 1 of 128 (0.8%) isolates carrying an MBL determinant (Table S7). The two discordant isolates, one KPC producer reported as resistant and one MBL producer reported as susceptible, were from patients who received other therapies. The eight isolates without available phenotypic results were all from patients treated with colistin or other agents. Amongthe 202 patients included in the weighted comparison, there was no discordance between enzyme class and phenotype; consequently, the pre-specified OXA-48/KPC sensitivity analysis also represented an analysis restricted to phenotypically confirmed susceptible infections.

Polymicrobial bloodstream infection was present in 71 patients (23.1%), most commonly involving another Enterobacterales species (*n* = 26), *Acinetobacter baumannii* (*n* = 20), *Candida* spp. (*n* = 13), Gram-positive cocci (*n* = 8), or *Pseudomonas aeruginosa* (*n* = 7). Polymicrobial episodes were distributed evenly across definitive regimen groups (*p* = 0.18), and 30-day mortality did not differ between polymicrobial and monomicrobial episodes (48/65, 73.8% versus 150/210, 71.4%, respectively; *p* = 0.75). Adding polymicrobial status to the PS model yielded a similar weighted estimate (OR 0.51; 95% CI 0.27–0.97). The estimate was also similar in the monomicrobial and polymicrobial strata, although imprecise in the latter (monomicrobial OR 0.53, 95% CI 0.25–1.13, *n* = 149; polymicrobial OR 0.45, 95% CI 0.14–1.47, *n* = 53). Crude mortality in the monomicrobial stratum was 63.9% (39/61) with C/AVI ± AZT versus 80.7% (71/88) with colistin, whereas, in the polymicrobial stratum, mortality was similar between the two treatment groups (72.0%, 18/25, versus 75.0%, 21/28, respectively) (Table S7, Panel E).

Patients without documented day-30 status (*n* = 33) had lower recorded markers of acute illness than those with a documented status: vasopressor requirement 15.2% versus 45.8% and ICU stay 36.4% versus 75.3%, respectively (both *p* < 0.001). An identifiable source of infection was also more frequent among patients with unknown status (*p* < 0.001). Age, CCI, carbapenemase class, viral pneumonia, and appropriateness of initial therapy did not differ between groups. Definitive treatment allocation (*p* = 0.84) and calendar period (*p* = 0.45) were comparable between the two groups (Table S10). Under the extreme assumption that all patients with unknown status survived, the weighted odds ratio for C/AVI ± AZT versus colistin was 0.57 (95% CI 0.32–0.98); under the assumption that all died, it was 0.56 (95% CI 0.30–1.04).

## 4. Discussion

### 4.1. Principal Findings

In this six-year, single-centre cohort of 308 adults with bloodstream infection caused by carbapenemase-producing Enterobacterales, 92.5% of isolates were *Klebsiella pneumoniae*, 44,1% carried an MBL determinant, and 31,8% carried dual carbapenemases. Overall 30-day mortality was 72% among the 275 patients with documented day-30 status, corresponding to 64.3% when calculated against the entire cohort. Vasopressor requirement and viral respiratory infection were the strongest independent predictors of death. Definitive therapy with ceftazidime/avibactam ± aztreonam (C/AVI ± AZT) was associated with lower 30-day mortality in the multivariable model (aOR 0.38; 95% CI 0.17–0.83) and in the IPTW-weighted comparison with colistin (OR 0.48; 95% CI 0.26–0.93), although the association was attenuated and no longer statistically significant after adjustment for calendar period (OR 0.72; 95% CI 0.37–1.42). Microbiological recurrence occurred in 10.1% of cases, was concentrated in the first days after the index blood culture, and was more frequent among patients infected with single OXA-48-like or MBL-producing isolates.

### 4.2. A Mortality Substantially Higher than in Most Published Cohorts

Our 30-day mortality was at the upper end of the published range and warrants consideration. A pooled analysis of carbapenem-resistant *K. pneumoniae* infections reported 42.1% mortality compared with 21.1% in carbapenem-susceptible controls [14], while contemporary CPE bloodstream infection cohorts from Latin America and France reported 30-day mortality of approximately 30–45% [15,16,17]. In a prospective Italian series restricted to MBL-producing Enterobacterales, 30-day mortality was 29.7% [18]. The multinational CRACKLE-2 cohort likewise demonstrated substantial regional variation in outcomes among patients infected with the same organisms, reflecting differences in patient characteristics, healthcare resources, and the intensity of supportive care [19].

Four features of our cohort may plausibly account for the higher mortality observed. First, eligibility required both an identified resistance mechanism and administration of antimicrobial therapy, thereby selecting for recognised, clinically significant infections while excluding milder or unrecognised episodes. Second, the case mix more closely resembles an intensive care population than a general bacteraemia cohort: the median age was 69 years, the median CCI was 6, 71.1% of patients were in critical care at the time of the index culture, 82.8% had an ICU stay, and 42.5% required vasopressors. Third, the carbapenemase distribution was unfavourable, with MBL determinants present in almost half the isolates during a period when aztreonam-containing regimens were not systematically available. Fourth, and most importantly, we report all-cause rather than attributable mortality. In the EURECA matched-cohort study, the hazard of death associated with carbapenem resistance decreased from 2.57 to 1.41 after adjustment for active therapy, source control, and illness severity [20], which indicatesthat a substantial proportion of the crude mortality observed in cohorts such as ours may reflect underlying host severity rather than the resistance phenotype itself. Our findings should therefore be interpreted as describing the prognosis of a severely ill population in a high-burden endemic setting rather than as an attributable effect of carbapenemase production.

These considerations also have implications for generalisability. The absolute mortality observed in our cohort is likely to be particularly relevant to critically ill patients with MBL-dominant CPE infections treated in an endemic setting and during a period that included pandemic surges. It should not be directly extrapolated to settings in which CPE bloodstream infection occurs predominantly outside intensive care, KPC producers predominate, or active therapy is routinely available from the outset. The comparative treatment estimates may be more generalisable in direction than in absolute magnitude.

A further limitation concerns the 33 patients (10.7%) excluded for whom day-30 vital status could not be established. If missing status was non-random—for example, if patients who were transferred or discharged early were more likely to survive—the mortality estimate of 72% among patients with documented status would be biased upwards. The alternative calculation of 64.3%, obtained by using the entire cohort as the denominator while implicitly treating patients with unknown status as survivors, therefore provides a useful lower-bound scenario rather than an estimate of true mortality. The formal comparison in Section 3.7 supports the possibility of such bias: patients with unknown status had substantially fewer markers of acute illness, with vasopressor requirement in 15.2% versus 45.8% and ICU stay in 36.4% versus 75.3%. Thus, the true 30-day mortality is plausibly lower than 72%, although its precise magnitude cannot be established from the available data. Because definitive treatment allocation and calendar period were comparable between patients with known and unknown status and because the extremecase analyses yielded weighted treatment estimates of 0.56 and 0.57, respectively, the differential ascertainment of day-30 status is unlikely to fully account for the observed treatment association.

### 4.3. Determinants of Death: Severity Dominates, with a Caveat

Vasopressor requirement had by far the strongest association with mortality (aOR 7.39; 95% CI 3.43–15.91), consistent with the INCREMENT-CPE score, in which severe sepsis or septic shock at presentation was the most heavily weighted predictor of early death in CPE bacteraemia [21]. Two observations temper a causal interpretation of this finding. First, vasopressor requirement was assessed at or after the index blood culture collection and may therefore lie on the causal pathway between infection, disease progression, and ineffective early therapy. Treating it as a baseline covariate may therefore constitute over-adjustment and attenuate the estimated association between treatment and mortality. Second, its association was weaker in the 72 h landmark model (aHR 1.28; 95% CI 0.86–1.89) than in the whole-cohort Cox model (aHR 1.54; 95% CI 1.12–2.12), consistent with haemodynamic instability exerting its greatest prognostic effect during the early course of infection. Co-isolation of carbapenem-resistant *Acinetobacter baumannii* was not independently associated with 30-day mortality but showed a borderline association with shorter survival (aHR 1.57; 95% CI 0.92–2.68; Table 6). This direction is consistent with the high mortality reported for this organism in critically ill patients [22,23,24]. The absence of a validated severity score, such as SOFA or the Pitt bacteraemia score, remains an important source of residual confounding. Prospective studies in comparable settings should therefore incorporate validated severity scores, including INCREMENT-CPE or Pitt scores where appropriate, to ensure more robust risk-adjusted comparisons.

Bacterial proliferation and progression to systemic invasion during or after severe viral respiratory illness lie at the intersection of impaired host defence and innate pathogenic traits [25]. Structural disruption of the alveolar and endothelial barriers, together with an immune response unable to clear microbial spread yet hyperactive to the point of self-harm, contributes to unrestrained dissemination through dysregulated permeability [26,27]. Viral respiratory infection was also consistently associated with poorer outcomes across the statistical approaches applied in this study. However, viral pneumonia was present in 20.1% of the cohort, including SARS-CoV-2 in 16.2%, and was concentrated in the pandemic years, which also coincided with peak CPE incidence, restricted access to novel β-lactam/β-lactamase-inhibitor combinations, and substantial strain on critical-care capacity. Calendar period was incorporated into the treatment models (Section 3.7), and the estimates for viral pneumonia were essentially unchanged after adjustment. Nevertheless, viral pneumonia and pandemic-era care were closely correlated in this cohort, making it difficult to separate the biological effect of viral respiratory infection from differences in case mix, treatment availability, and healthcare delivery during the pandemic. Accordingly, this association should be interpreted as hypothesis-generating rather than as evidence of a specific biological interaction.

### 4.4. Ceftazidime/Avibactam Versus Colistin: What the Estimates Support, and What They Do Not

Novel β-lactam/β-lactamase-inhibitor combinations are guideline-endorsed treatment options for KPC- and OXA-48-producing Enterobacterales [6]. A Korean cohort of 292 patients with KPC-producing bloodstream infection reported improved survival with ceftazidime/avibactam compared with conventional regimens [7], while a French multicentre cohort and meta-analysis reported similar findings for OXA-48-producing infections [28]. In our study, the association between C/AVI ± AZT and lower mortality was directionally consistent across three analytical approaches: the crude head-to-head odds ratio versus colistin was approximately 0.51, the IPTW-weighted estimate was 0.48, and the fully adjusted multivariable estimate was 0.38. The similarity of the crude and IPTW estimates suggests that adjustment for the measured covariates did not materially alter the treatment association, whereas the stronger association in the multivariable model may reflect residual differences in baseline or acute illness severity between treatment groups. However, these analyses cannot exclude unmeasured confounding by indication. Notably, in univariate analysis, C/AVI ± AZT versus all other regimens was not significantly associated with mortality (OR 0.66; 95% CI 0.38–1.16), whereas colistin was associated with higher mortality (OR 1.92; 95% CI 1.10–3.35).

This asymmetry is important for interpreting the treatment comparison. Thirty-day mortality was 66.3% (57/86) with C/AVI ± AZT, 79.3% (92/116) with colistin, and 67.1% (49/73) with other regimens. The similar mortality observed in the C/AVI ± AZT and other therapy groups indicates that the data are compatible with both a benefit associated with C/AVI ± AZT and poorer outcomes among patients selected for colistin. The latter interpretation is biologically plausible given the unfavourable pharmacokinetics and nephrotoxicity of polymyxins and the outcome signals reported in previous studies [10,11,12]. Consistent with this, a meta-analysis found lower nephrotoxicity with ceftazidime/avibactam than with comparator regimens (RR 0.41; 95% CI 0.20–0.84) [29]. The absolute difference in crude mortality between C/AVI ± AZT and colistin was 13 percentage points, although this unadjusted difference should not be interpreted as a treatment effect because treatment allocation was non-randomised. The magnitude of the observed association is nevertheless broadly consistent with the pooled 30-day mortality benefit reported for ceftazidime/avibactam in KPC-producing bloodstream infection (RR 0.59; 95% CI 0.46–0.75) [29] and with findings from cohorts of transplant recipients, patients with haematological malignancies, nosocomial *Klebsiella pneumoniae* infection, and post-surgical pneumonia [30,31,32,33,34,35,36].

The pre-specified sensitivity analysis restricted to OXA-48-/KPC-producing isolates yielded an essentially unchanged point estimate (OR 0.49; 95% CI 0.20–1.19 versus 0.48; 95% CI 0.26–0.93, respectively), although the confidence interval widened as the sample decreased from 202 to 110 patients. The loss of statistical significance therefore reflects reduced precision rather than a diminished treatment effect [37]. Carbapenemase class was confirmed in every isolate by two independent methods, whereas phenotypic susceptibility to ceftazidime/avibactam was not systematically available at the time of treatment. The subgroup was therefore initially defined by enzyme class rather than phenotype. This distinction is relevant because OXA-48- or KPC-producing isolates may nevertheless be non-susceptible to ceftazidime/avibactam because of additional resistance mechanisms, including porin loss, efflux upregulation, or the overproduction of ESBLs or AmpC enzymes. Non-susceptibility among carbapenem-resistant *K. pneumoniae* has also been reported at high frequency in Romanian tertiary centres [38].

The phenotypic data available in our cohort substantially reduce this concern. Across the cohort, 99.4% of isolates producing KPC or OXA-48-like enzymes without an MBL determinant were phenotypically susceptible, and all isolates assigned to the OXA-48/KPC stratum among the 202 patients included in the weighted comparison were susceptible (Section 3.7). Thus, within the weighted analysis, phenotype is unlikely to explain the loss of statistical significance in the OXA-48/KPC subgroup, which is more plausibly attributable to the smaller sample and consequent imprecision. The higher non-susceptibility reported from other Romanian centres [38] may partly reflect the inclusion of MBL co-producers, which were uniformly non-susceptible in our series. This distinction is important when extrapolating those data to our cohort. Colistin remained the sole comparator in the primary treatment analysis.

The most consequential finding of the additional analyses concerned calendar time, and it requires emphasis rather than qualification. When calendar period was added to both the PS and weighted outcome models, the association between C/AVI ± AZT and 30-day mortality was attenuated from OR 0.48 to 0.72 (95% CI 0.37–1.42) and lost statistical significance. Similarly, the fully adjusted multivariable estimate changed from aOR 0.39 to 0.44 (95% CI 0.19–1.02). The primary treatment association was therefore not robust to adjustment for calendar period.

Two non-mutually exclusive explanations are possible. First, calendar period may represent residual confounding by changes in patient characteristics, treatment availability, critical-care capacity, or other aspects of clinical management over time. Ceftazidime/avibactam became increasingly available during the study period, while the final epoch also differed substantially in carbapenemase distribution and healthcare context. Second, the association between treatment and outcome may have differed according to the clinical context in which C/AVI ± AZT was used. Crude 30-day mortality among patients receiving colistin remained relatively stable across the three epochs (81.1%, 76.6%, and 81.2%), whereas mortality among those receiving C/AVI ± AZT decreased from 83.3% to 72.2% and 60.7%. This temporal pattern is compatible with increasing effectiveness as C/AVI ± AZT became more routinely available, but it cannot distinguish a treatment effect from concurrent changes in case mix and clinical management. In particular, only 12 patients received C/AVI ± AZT in the first epoch, which makes the corresponding estimate highly imprecise. The epoch-specific findings should therefore be regarded as hypothesis-generating rather than as evidence of effect modification by calendar period.

Pooling ceftazidime/avibactam alone with ceftazidime/avibactam plus aztreonam also requires caution because these two regimens are not interchangeable. Avibactam does not inhibit MBLs; consequently, ceftazidime/avibactam alone is not expected to provide reliable activity against isolates carrying an MBL determinant, whereas the addition of aztreonam can restore activity by protecting aztreonam from co-produced serine β-lactamases. Of the 98 patients treated with C/AVI ± AZT, 34 received the combination, all of whom had MBL-carrying isolates; the remaining 16 patients with MBL-producing isolates received ceftazidime/avibactam alone or in combination with colistin. The similar weighted estimates in the MBL and OXA-48/KPC strata (OR 0.46 and 0.48, respectively) should therefore not be interpreted as evidence that ceftazidime/avibactam itself is effective against MBL-producing organisms.

Rather, the MBL estimate reflects a heterogeneous set of C/AVI-based regimens, including aztreonam-containing therapy, and remains susceptible to residual confounding.

The regimen-specific analysis provides some additional context. Relative to colistin-based therapy, lower odds of death were observed with ceftazidime/avibactam alone (OR 0.27; 95% CI 0.09–0.82) and ceftazidime/avibactam plus aztreonam (OR 0.09, 95% CI 0.02–0.43), whereas the corresponding estimates for ceftazidime/avibactam plus colistin (OR 0.96, 95% CI 0.33–2.81) and ceftazidime/avibactam plus both agents (OR 0.50; 95% CI 0.12–2.05) were less pronounced and imprecise. The aztreonam-containing estimate, which was based on only 12 patients with documented day-30 status, should therefore be interpreted cautiously despite remaining significant after Firth penalisation.

The concentration of the association in colistin-free regimens is compatible with poorer outcomes among patients receiving colistin, but confounding by indication remains a plausible explanation because colistin may have been preferentially continued or added in patients with clinical deterioration or limited expected antimicrobial options. These data therefore support the plausibility of a role for active C/AVI-based therapy but do not establish the superiority of any individual regimen.

Polymicrobial bloodstream infection, present in 23.1% of episodes, did not materially confound the primary treatment comparison: its distribution across treatment groups was similar, and inclusion in the PS score yielded a comparable weighted estimate (OR 0.51; 95% CI, 0.27–0.97). The association was more apparent in monomicrobial episodes than in polymicrobial episodes, although the latter subgroup was small and the confidence interval was wide. This pattern is biologically plausible because outcomes in polymicrobial infection may be influenced by pathogens and therapeutic requirements beyond those of the *Enterobacterales* isolate. However, this subgroup finding should be considered exploratory.

### 4.5. MBL and Dual-Carbapenemase Producers: The Widest Evidence Gap

Our carbapenemase distribution—with MBL determinants present in 44.1% of isolates and dual carbapenemases in 31.8%—differs substantially from that reported in various CPE series but is consistent with the epidemiology described in the region. A contemporaneous Bucharest cohort of 340 carbapenem-resistant *K. pneumoniae* isolates found double NDM plus OXA-48-like co-production in 53.6%, with resistance rates exceeding 70% to ceftazidime/avibactam, ceftolozane/tazobactam, imipenem/relebactam, and colistin and 32.1% to cefiderocol [38]. A comparable predominance of MBL and OXA-48-like co-producers has been reported in other Romanian hospitals [39], mirroring the post-pandemic epidemiological shift described in Greece [2] and consistent with reports from the Middle East and North Africa [3,4,40,41]. MBL-producing Enterobacterales are particularly challenging because many species carrying these enzymes also have an intrinsic resistance to important last-line agents, including colistin and tigecycline, as exemplified by *Providencia* spp., *Proteus* spp. and *Serratia* spp. [42,43,44]. At the population level, the ECDC estimated that the incidence of carbapenem-resistant *K. pneumoniae* bloodstream infection in the EU/EEA rose by more than 60% between 2019 and 2024, with the highest burden in southern, central and eastern Europe [45].

Thirty-day mortality among patients with MBL-producing infections was 70.6% (89/126) in our cohort, substantially higher than the 29.7% reported by Falcone and colleagues [18]. Several differences may account for this disparity. Their series included infection from all anatomical sites rather than bloodstream infection alone; ceftazidime/avibactam plus aztreonam was systematically available as the first-line therapy, with early active treatment independently associated with lower mortality; and synergy testing was used to guide the combination. In our cohort, aztreonam-containing regimens were not consistently available, and only seven patients received an alternative MBL-directed strategy (cefiderocol, *n* = 3; meropenem–ertapenem, *n* = 4). This limited availability of active MBL-directed therapy represents an important treatment system constraint and an actionable target for improving outcomes in comparable settings. Meta-analytic data support the use of aztreonam-based combinations, with a pooled 30-day mortality risk ratio of 0.51 (95% CI 0.34–0.76) compared with polymyxins in MBL-producing CRE bloodstream infection [46], while the ASSEMBLE trial provides randomised evidence supporting aztreonam–avibactam [8]. Cefiderocol remains an alternative for selected infections caused by NDM- and VIM-producing organisms, although comparative clinical outcome data remain limited [9,47,48,49]. Importantly, the fixed aztreonam–avibactam product and the off-label combination of ceftazidime/avibactam with aztreonam used in our cohort [50,51] are not pharmacologically interchangeable; our findings therefore apply only to the latter regimen.

**Figure 5:**
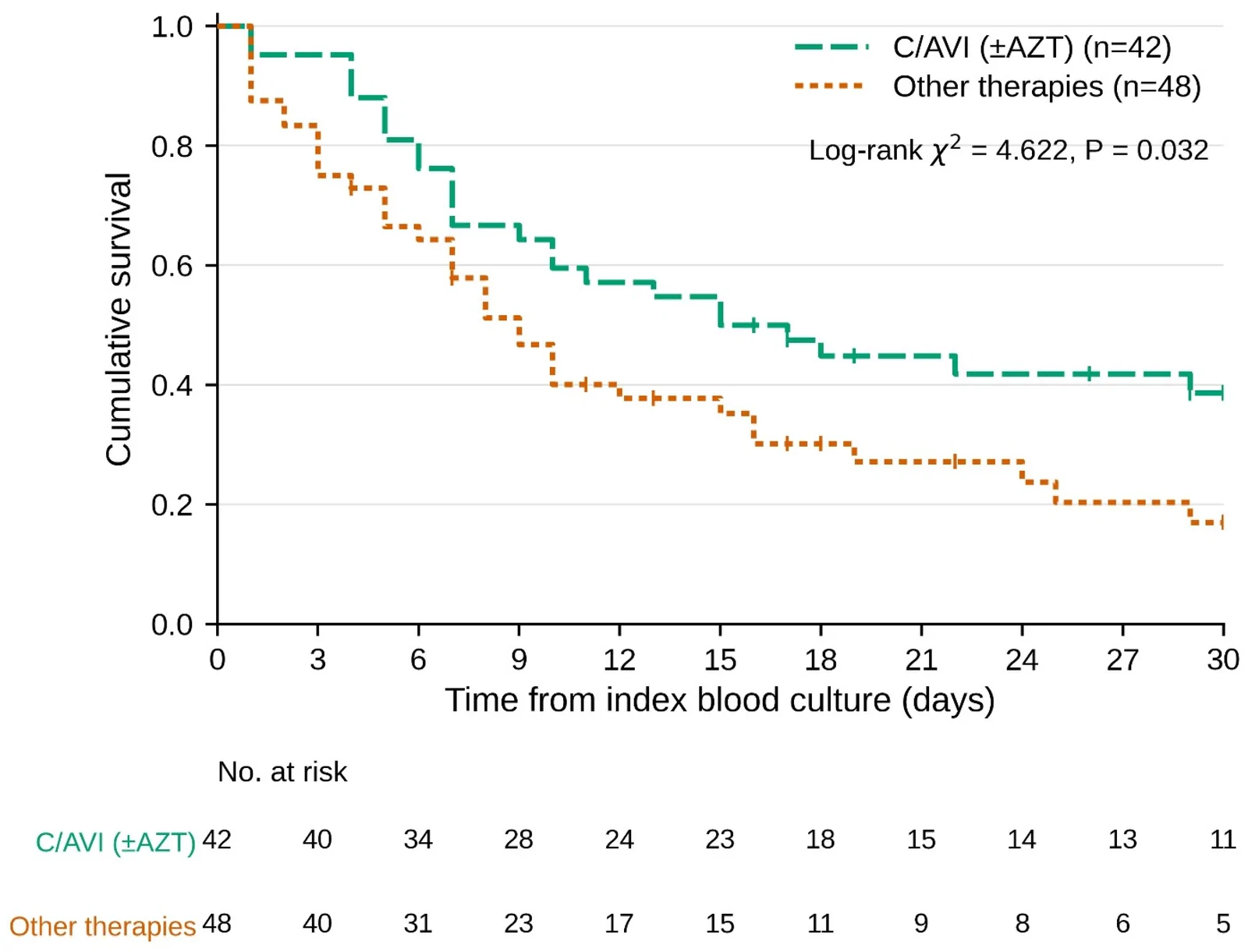
Kaplan–Meier estimates of 30-day survival by definitive treatment in patients infected with dual carbapenemase producers co-harbouring NDM (OXA-48/KPC + NDM *n* = 90, C/AVI ± AZT *n* = 42, other therapies *n* = 48; log-rank χ^2^ = 4.622; *p* = 0.032). AZT aztreonam, C/AVI ceftazidime/avibactam.

**Figure 6:**
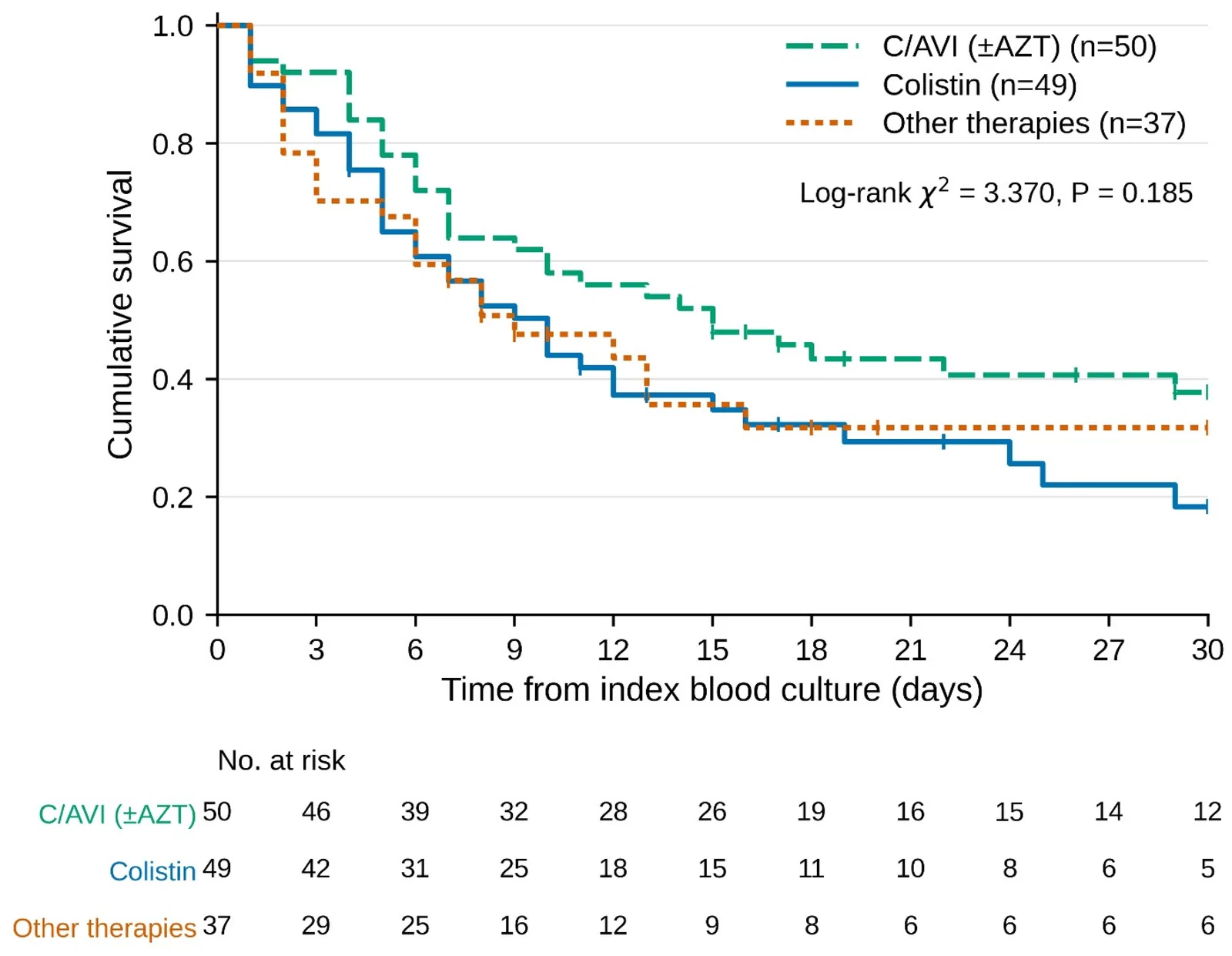
Kaplan–Meier estimates of 30-day survival by definitive treatment in patients infected with metallo-β-lactamase-producing isolates (*n* = 136, C/AVI ± AZT *n* = 50, colistin *n* = 49, other therapies *n* = 37; log-rank χ^2^ = 3.370; *p* = 0.185). AZT aztreonam, C/AVI ceftazidime/avibactam.

Unadjusted Kaplan–Meier survival curves favoured C/AVI ± AZT over other definitive regimens among the 90 patients infected with dual carbapenemase producers co-harbouring NDM (Figure 5; χ^2^ = 4.622; *p* = 0.032), whereas no significant difference in survival across definitive-treatment strata was observed among the broader group of 136 patients carrying an MBL determinant (Figure 6; χ^2^ = 3.370; *p* = 0.185). Given the small subgroup sizes, the lack of adjustment, and the potential for substantial confounding by indication, these findings should be considered exploratory and should not be interpreted as evidence of comparative treatment efficacy in MBL-producing infections.

### 4.6. Microbiological Recurrence

Recurrence occurred in 10.1% of patients after a median of four days (IQR 2–6). This short interval between the index and follow-up cultures is compatible with microbiological persistence rather than true relapse, although these entities cannot be formally distinguished without molecular typing. The carbapenemase gene was confirmed in both index and follow-up isolates, but sequence typing was not performed. Consequently, misclassification remains possible in both directions: an unrelated isolate of the same species carrying the same carbapenemase gene—an entirely plausible event in a setting with substantial colonisation by carbapenem-resistant Enterobacterales—would be classified as persistence, whereas a genuine relapse involving a variant of the index strain that had acquired or lost a resistance determinant could be missed. The recurrence rate should therefore be interpreted as an operational composite endpoint rather than as a measure of true microbiological relapse.

Single OXA-48-like or MBL producers were more likely to be recovered in follow-up culture than isolates in the aggregated reference group comprising KPC producers and dual producers(aOR 2.46; 95% CI 1.12–5.41), a finding partially consistent with an international multicentre study reporting more frequent recurrence among NDM producers than among KPC producers [52]. However, the aggregation of biologically distinct carbapenemase groups into a single reference category limits the interpretation of this association, and future analyses should evaluate each carbapenemase class separately. A shorter interval from hospital admission to the index bloodstream infection was independently associated with recurrence (aOR 0.94 per day; 95% CI 0.90–0.98). Rather than representing a microbiological characteristic, this association may reflect the earlier development of the bloodstream infection during hospitalisation and a potentially unresolved infection or incomplete source control. The higher frequency of recurrence among patients receiving corticosteroids at the onset of infection (13.9% vs. 7.5%; *p* = 0.08) did not reach statistical significance and should therefore be regarded as hypothesis-generating only.

### 4.7. Empirical Therapy and Source Control

Inappropriate initial therapy was common (76%), and 98.7% of patients had recent antibiotic exposure. The absence of an observed association between inadequate empirical therapy and mortality is therefore most plausibly explained by the limited contrast between treatment groups and consequent low statistical power than by a genuine absence of effect. In the INCREMENT cohort, inappropriate early targeted therapy was independently associated with mortality (OR 2.47; 95% CI 1.58–4.63) [21], and early active therapy reduced mortality in MBL-producing infection [18]. Because the exact interval from index blood culture collection to administration of the first active antimicrobial could not be reconstructed from the prescription records, we modelled initial and definitive therapy separately and could not directly evaluate the effect of time to appropriate therapy. We consider this an important source of residual confounding in the treatment comparison rather than merely a descriptive limitation.

With 76% of patients initially receiving inactive empirical therapy, the time to effective treatment likelyvaried substantially; carbapenemase characterisation, the decision to escalate to a novel β-lactam/β-lactamase-inhibitor combination, and administration of an active agent may occur within a short interval; consequently, patients who received C/AVI ± AZT may have achieved effective therapy earlier than those who remained on colistin, and part of the observed difference in survival may therefore reflect treatment timing rather than the specific antimicrobial regimen. The 72 h landmark analysis mitigates immortal-time bias but does not eliminate this confounding.

The one treatment-timing variable that could be reconstructed did not resolve this issue. The interval from index blood culture collection to initiation of ceftazidime/avibactam was not associated with 30-day mortality, either when analysed categorically (61.1%, 69.2% and 73.5% for 0–1, 2 and more than 2 days, respectively; *p* = 0.54) or continuously (adjusted OR 1.12 per additional day; 95% CI 0.85–1.46). This null result, however, was based on only 83 patients and does not exclude an effect of time to first active antimicrobial, which is a different and unmeasured variable. The latter applies to both treatment groups, whereas the interval to ceftazidime/avibactam initiation can only be assessed among patients who received C/AVI-based therapy. Prospective studies in this setting should therefore record the time from bloodculture positivity to the first active antimicrobial as a pre-specified covariate.

One aspect of our workflow may have limited the extent of differential delay between treatment groups. Carbapenemase class was established by rapid immunoassay and multiplex PCR on positive blood cultures, which madethe information needed to select an active regimen available within hours of culture positivity for all patients. The potential for prolonged differential delay was therefore probably lower than in settings in which treatment decisions depend on later phenotypic confirmation. In a setting where three-quarters of patients receive inactive empirical treatment and MBL determinants are present in nearly half of isolates, rapid carbapenemase characterisation directly from the positive blood culture is the intervention that is most likely to change the outcome [6,38]. Source control is another key determinant of successful treatment [53], although no focus-related variable was independently associated with microbiological clearance in our cohort.

### 4.8. Strengths and Limitations

The strengths of this study are its size for a single-centre cohort, the six-year continuous inclusion period, the unusually high representation of MBL and dual-carbapenemase producers, and the confirmation of carbapenemase class in every isolate by two independent methods—rapid immunoassay and multiplex PCR—which permitted reliable stratification by enzyme class, including identification of dual producers, in contrast to cohorts relying on phenotypic inference alone. Colistin susceptibility was determined by broth microdilution in accordance with EUCAST recommendations, avoiding the misclassification associated with automated polymyxin testing. Complementary analytical strategies—multivariable regression, PS weighting and a 72 h landmark analysis—were used to address confounding by indication and immortal-time bias.

Several limitations should be considered. The design is single-centre and retrospective, precluding control of unmeasured confounding; the results reflect an endemic, MBL-dominant, critically ill population and should not be extrapolated to settings with different carbapenemase epidemiology. Ceftazidime/avibactam susceptibility was not included in the routine extended panel throughout the study period. However, a phenotypic result was retrievable for 300 of the 308 isolates and confirmed susceptibility in every isolate assigned to the OXA-48/KPC stratum of the weighted comparison; misclassification of treatment eligibility within this subgroup is therefore unlikely to be a major limitation, although the eight isolates without an available result and the absence of routine testing in the earlier years remain limitations. Minimum inhibitory concentrations were not retrieved in a standardised form for all isolates and were therefore not analysed as continuous exposures, which precludedany assessment of MIC–outcome relationships. Sequence typing and whole-genome sequencing were not undertaken, so clonal relatedness could not be assessed, and microbial persistence could not be formally distinguished from true relapse. Validated severity scores were not consistently recorded, and the surrogates used (vasopressor requirement, critical-care admission) may function partly as mediators rather than baseline confounders. Calendar period has been incorporated into the analyses and has attenuated the treatment association. Because the epochs differed simultaneously in drug availability, case mix, critical-care strain, and carbapenemase distribution, and because C/AVI ± AZT and colistin were unevenly distributed across periods, temporal confounding cannot be disentangled from other changes in clinical practice or outcome risk. The regimen-specific estimates were based on small groups, including one analysis with only 12 patients with documented day-30 status, and were not derived from PS models; they should therefore be regarded as exploratory despite adjustment for selected covariates. The interval to initiation of ceftazidime/avibactam was defined only among treated patients and is not a substitute for the time to first active antimicrobial, which was unavailable. Doses, infusion durations, and renal adjustments were documented in free-text prescription notes rather than structured fields and could not be abstracted reliably for every patient; therapeutic drug monitoring of colistin is not performed at our institution. Therefore, systematic underexposure to colistin, a recognised driver of failure with this agent, cannot be excluded and may have contributed to the difference observed between treatment groups. Day-30 vital status was undocumented in 10.7% of patients. The MBL, dual-producer, and aztreonam-treated subgroups were relatively small, which resulted in imprecise estimates in several sensitivity analyses. The non-significant sensitivity analyses should therefore not be interpreted as evidence of no association. Colistin was the only comparator in the primary treatment analysis, and some carbapenemase reference categories were aggregated because of sample size. Finally, mortality was an all-cause endpoint; cause of death was not adjudicated, and infection-related death could not be separated from death due to underlying disease. We did not attempt retrospective attribution because such adjudication would be inherently uncertain in a population with substantial comorbidity (median CCI, 6) and severe acute illness (42.5% requiring vasopressors). Consequently, the treatment comparison was based on an endpoint that includes deaths that may not have been preventable by antimicrobial treatment alone. This, together with the high baseline event rate, may limit the extent to which differences in antimicrobial effectiveness can be reflected in all-cause mortality.

Taken together, these limitations mean that the observed treatment differences should be interpreted as associations under routine clinical care rather than as estimates of a causal treatment effect.

## 5. Conclusions

In this high-mortality cohort of carbapenemase-producing Enterobacterales bloodstream infections, dominated by *Klebsiella pneumoniae*, definitive therapy with ceftazidime/avibactam ± aztreonam was associated with lower 30-day mortality than colistin, with a directionally consistent association across crude, propensity-weighted, and fully adjusted analyses.However, this association was attenuated after adjustment for calendar period, did not reach significance in the pre-specified OXA-48/KPC sensitivity analysis, and was most apparent during the final two years of the study, when ceftazidime/avibactam was routinely available and could be started earlier. Mortality among patients receiving colistin remained relatively stable across the study period. The retrospective, non-randomised design; absence of validated severity scores; and inability to reconstruct the timeto first active antimicrobial preclude inference of a causal survival benefit.

Because mortality among patients receiving other active regimens was similar to that observed with C/AVI ± AZT, our findings are compatible with both a benefit associated with C/AVI ± AZT and poorer outcomes among patients selected for colistin-based therapy. The data therefore support preferential use of active, non-colistin-based regimens when appropriate rather than establishing an agent-specific survival advantage. Vasopressor requirement and viral respiratory co-infection identified patients at particularly high risk of death. In a setting where MBL determinants were present in nearly half of isolates and three-quarters of patients initially received inactive empirical treatment, rapid carbapenemase characterisation directly from positive blood cultures and reliable access to aztreonam-containing regimens represent important priorities for earlier optimisation of antimicrobial therapy. Prospective, adequately powered multicentre studies incorporating phenotypic susceptibility testing, validated severity scores, regimen-specific exposures, and time to first active antimicrobial therapy are required before causal comparative effectiveness can be established.

## Figures and Tables

**Table 1:** Standardised mean difference for variables used in logistic regression for treatment before and after weighting in patients receiving colistin or ceftazidime/avibactam (+/−) aztreonam, in patients infected with OXA-48-/KPC-producing Enterobacterales, respectively.

	Before	After
Analysis on cohort (202)		
Age	0.09	−0.005
Charlson score	0.19	0.0025
Metallo beta lactamase present	0.29	0.002
Vasopressor	0.05	0.017
ICU admission	0.04	0.0015
Unidentified source	−0.062	0.0033
Central line	0.1599	0.003
Urinary	−0.039	−0.017
Respiratory	0.048	−0.013
Other (skin, soft tissues, intra-abdominal)	0.0300	0.03
Sensitivity analysis for OXA48/KPC/KPC + OXA48 (110)		
Age	0.09	0.0504
Charlson score	0.104	0.042
Vasopressor	0.07	−0.016
Intensive care unit	0.21	0.035
Unidentified source	−0.031	−0.0314
Central line	0.071	0.013
Urinary	−0.235	0.049
Respiratory	0.1807	0.041

**Table 2:** Demographic, clinical, and microbiological characteristics and laboratory findings of patients with BSI caused by carbapenemase-producing Enterobacterales (*N* = 308).

Feature	Value
Male sex, *n* (%)	175 (56.8%)
Age, median (IQR)	69 (58–77)
Number of in-hospital days, median (IQR)	27 (15–44)
Charlson comorbidity index, median (IQR)	6 (4–8)
Admission from	
Hospital, *n* (%)	60 (19.5%)
Long-term care facilities, *n* (%)	46 (14.9%)
Community, *n* (%)	202 (65.6%)
Number of ICU days, median (IQR)	12 (4–25)
Number of days from hospital admission to bacteraemia, median (IQR)	14 (8–25)
Days to microbial recurrence, median (IQR)	4 (2–6)
History of smoking, *n* (%)	68 (22.1%)
Alcohol consumption, *n* (%)	48 (15.6%)
Recent surgical interventions, *n* (%)	113 (36.7%)
Intra-abdominal surgery, *n*(%)	32 (10.4%)
Cranial traumatism, *n* (%)	24 (7.8%)
Active smoker, *n* (%)	41 (13.3%)
Chronic and obstructive respiratory disease, *n* (%)	45 (14.6%)
Diabetes mellitus, *n* (%)	105 (34.1%)
Thyroid gland disorders, *n* (%)	25 (8.1%)
Chronic kidney disease, *n* (%)	70 (22.7%)
Ulcers or GI bleeding history, *n* (%)	40 (13%)
Malignancies, *n* (%)	63 (20.5%)
Recorded or suspected secondary tumors, *n* (%)	35 (11.4%)
Haematologic malignancy, *n* (%)	6 (1.94%)
Localised tumor, *n* (%)	30 (9.7%)
Neurocognitive disorders (dementia), *n* (%)	85 (27.6%)
Cerebrovascular disease, *n* (%)	122 (39.6%)
Intracranial haemorrhage, *n* (%)	44 (14.3%)
Cardiovascular disease, *n* (%)	195 (63.3%)
Chronic heart failure, *n* (%)	103 (33.4%)
Coronary heart disease, *n* (%)	76 (24.7%)
Myocardial infarction, *n* (%)	33 (10.7%)
Peripheral artery disease, *n* (%)	28 (9.1%)
Obesity, *n* (%)	130 (42.2%)
Liver disease, *n* (%)	39 (12.7%)
Use of vasopressors, *n* (%)	131 (42.5%)
Use of hypotensors, *n* (%)	58 (18.8%)
Necessity of systemic steroids, *n* (%)	122 (39.6%)
Critical-care stay at time or after bacteraemia, *n* (%)	219 (71.1%)
Immunosuppression, *n* (%)	104 (33.8%)
Prior corticosteroid use, *n* (%)	78 (25.3%)
Prior chemotherapy, *n* (%)	26 (8.4%)
Passage through an ICU, *n* (%)	255 (82.8%)
Viral pneumonia, *n* (%)	71 (20.1%)
SARS-CoV-2, *n* (%)	50 (16.2%)
Influenza, *n* (%)	15 (4.9%)
Suspected or unconfirmed viral pneumonia, *n* (%)	6 (1.94%)
*Clostridium* sp.during hospitalisation, *n* (%)	31 (10.1%)
Type of carbapenemase	
OXA48/KPC + NDM, *n* (%)	90 (29.2%)
MBL, *n* (%)	46 (14.9%)
KPC/KPC + OXA48, *n* (%)	101 (32.8%)
OXA48, *n* (%)	71 (23.1%)
Prior colonisation with carbapenem-resistant Enterobacterales, *n* (%)	154 (50%)
Same carbapenemase expressed in colonisationinfection (among colonised patients, *N* = 154), *n* (%)	97 (63%)
Source of BSI	
Urinary source, *n* (%)	49 (15.9%)
Catheter-related (central line-related), *n* (%)	24 (7.8%)
Respiratory source, *n* (%)	33 (10.7%)
Other (biliary, cutaneous, soft tissues), *n* (%)	7 (2.3%)
Unidentified source, *n* (%)	195 (63.3%)
Polymicrobial BSI, *n* (%)	71 (23.1%)
*Pseudomonas aeruginosa* co-isolation, *n* (%)	7 (2.3%)
*Acinetobacter baumannii* co-isolation, *n* (%)	20 (6.5%)
Enterobacteriaceaeco-isolation, *n* (%)	26 (8.4%)
*Candida* sp. co-isolation, *n* (%)	13 (4.2%)
Cocci+ co-isolation, *n* (%)	8 (2.6%)
Exposure to	
Carbapenems, *n* (%)	133 (43.2%)
Cephalosporins, *n* (%)	109 (35.4%)
Piperacillin/tazobactam, *n* (%)	77 (25.2%)
Penicillins, *n* (%)	22 (7.14%)
Colistin, *n* (%)	76 (24.7%)
Fluoroquinolones, *n* (%)	62 (20.1%)
Vancomycin, *n* (%)	40 (13%)
Linezolid, *n* (%)	56 (18.2%)
Aminoglycosides, *n* (%)	10 (3.2%)
Clindamycin, *n* (%)	21 (6.8%)
Metronidazole, *n* (%)	38 (12.3%)
Tigecycline, *n* (%)	12 (3.9%)
In-hospital mortality	217 (70.5%)
7-day mortality, *n* (%), *N* = 303	104 (34.32%)
14-day mortality, *n* (%), *N* = 297	163 (54.88%)
21-day mortality, *n* (%), *N* = 287	185 (64.45%)
30-day mortality, *n* (%), *N* = 275	198 (72%)
Bacterial recurrence, *n* (%)	31 (10.1%)
Initial therapy	
Inadequate initial therapy, *n* (%)	234 (76%)
C/AVI (+/−AZT), *n* (%)	32(10.38%)
Colistin, *n* (%)	98 (31.8%)
Other antimicrobials, *n* (%)	178 (57.82%)
Antimicrobials associated	
Fluoroquinolones, *n* (%)	21 (6.8%)
Tigecycline, *n* (%)	20 (6.5%)
Aminoglycosides, *n* (%)	6 (1.9%)
Definitive therapy	
C/AVI (+/−AZT), *n* (%)	98 (31.8%)
Colistin, *n* (%)	129 (41.9%)
Other antimicrobials, *n* (%)	81 (26.3%)
Laboratory findings	
Parameter (unity of measurement)	Value
Fibrinogen (mg/dL), median (IQR), *N* = 288	441 (348–540)
Albumin (g/dL), median (IQR), *N* = 217	2.6 (2.2–2.9)
Leukocytes(10^3^/μL), median (IQR)	12.7 (8.71–18.4)
Lymphocytes (10^3^/μL), median (IQR)	0.78 (0.44–1.28)
Monocytes 10^3^/μL, median (IQR)	0.53 (0.26–0.83)
Neutrophils (10^3^/μL),median (IQR)	11 (7.24–16.7)
Erythrocytes (10^6^/μL), median (IQR)	3.21 (2.76–3.73)
Thrombocytes (10^3^/μL), median (IQR), *N* = 307	196 (125–267)
Urea (mg/dL), median (IQR)	64 (38–103)
Creatinine (mg/dL), median (IQR), *N* = 307	0.9 (0.6–1.7)
Glucose (mg/dL) median (IQR), *N* = 305	111 (86–146)
Potassium (meq/mol), median (IQR), *N* = 308	3.8 (3.4–4.3)
Sodium (meq/mol), median (IQR), *N* = 308	140 (136–145)
D-Dimers (ng/mL), median (IQR) *N* = 207	1389 (754–2824)
Haemoglobin (g/dL), median (IQR)	9.3 (8–10.8)
C Reactive Protein (mg/L), median (IQR), *N* = 293	158 (75.6–240)
Procalcitonin (ng/mL), median (IQR), *N* = 303	3.57 (0.79–16.1)
ALT (U/L), median (IQR), *N* = 293	30 (17–65)
AST (U/L), median (IQR), *N* = 293	42 (28–78)

AZT: aztreonam; ALT: Alanine Aminotransferase; AST: Aspartate Aminotransferase; BSI:bloodstream infection; GI: gastrointestinal; ICU: intensive care unit; IQR: interquartile range; C/AVI: ceftazidime/avibactam; CI95%: confidence interval 95%; CR: carbapenem-resistant; MBL: metallo beta lactamase.

**Table 3:** Multivariate analysis of factors associated with 30-day mortality in bloodstream infections caused by carbapenemase-producing Enterobacterales.

Variable	B	S.E.	WALD	*p*	aOR (CI95%)
Age	0.021	0.014	2.412	0.12	1.02 (0.99–1.05)
Charlson comorbidity index	0.094	0.074	1.616	0.204	1.09 (0.95–1.27)
Active smoker	−0.86	0.451	3.635	0.057	0.42 (0.17–1.02)
Viral pneumonia	1.272	0.507	6.291	0.012 *	3.56 (1.32–9.63)
Bacterial pneumonia	0.755	0.368	4.22	0.04 *	2.12 (1.03–4.38)
Vasopressor necessity	2.001	0.391	26.217	0.000 *	7.39 (3.43–15.91)
Source of BSI (No source Ref.)			2.633	0.45	
Catheter	−0.926	0.617	2.251	0.13	0.39 (0.11–1.32)
Respiratory	0.292	0.652	0.201	0.65	1.34 (0.37–4.8)
Urinary, biliary, soft tissue	−0.045	0.439	0.01	0.91	0.95 (0.4–2.26)
Inadequate initial therapy	0.551	0.394	1.953	0.16	1.73 (0.8–3.75)
Type of carbapenemase (single MBL as REF)			2.59	0.459	
KPC	0.485	0.493	0.966	0.32	1.62 (0.61–4.26)
OXA48	0.061	0.516	0.014	0.9	1.06 (0.38–2.92)
OXA48 + NDM	0.639	0.499	1.642	0.2	1.89 (0.71–5.03)
Definitive therapy (Colistin therapy as REF)			6.486	0.039 *	
C/AVI(+/−AZT)	−0.962	0.4	5.771	0.016 *	0.38 (0.17–0.83)
Others	−0.762	0.423	3.245	0.072	0.46 (0.2–1.06)
Constant	−2.507	1.008	6.19	0.01	0.08

* *p* < 0.05; aOR: adjusted odds ratio; AZT: aztreonam; BSI: bloodstream infection; C/AVI: ceftazidime/avibactam;CI95%: confidence interval 95%; MBL: metallo beta lactamase; REF: Reference.

**Table 4:** Univariate analysis of demographic, clinical and laboratory variables in relation to recurrent or persistent bacteraemia.

Feature	Event Yes (*N* = 31)	Event No (*N* = 277)	*p*	OR CI95%
Age, median (IQR)	62 (54.5–74)	69 (58–77)	0.203	0.99 (0.58–1.65)
CCI, median (IQR)	5 (3–7)	6 (4–8)	0.057	1.02 (0.94–1.11)
Male sex, *n* (%)	20 (64.5%)	155 (56%)	0.36	1.4 (0.66–3.1)
Length of stay, median (IQR)	14 (8–31)	27 (14–45)	0.482	
Number of days until bacteraemia, median (IQR)	10 (4–16)	15 (8–26)	0.007 *	
ICU length of stay, median (IQR)	9 (3–19)	13 (7–29)	0.056	
History of smoking, *n* (%)	9 (29%)	59 (21.3%)	0.74	1.51 (0.66–3.45)
Active smoker, *n* (%)	4 (12.9%)	37 (13.4%)	0.94	0.96 (0.31–2.9)
Chronic alcohol consumption, *n* (%)	7 (22.6%)	41 (14.8%)	0.29	1.67 (0.67–4.15)
Recent surgical interventions, *n* (%)	14 (45.2%)	99 (35.7%)	0.3	1.48 (0.7–3.13)
ICU stay, *n* (%)	23 (74.2%)	196 (70.8%)	0.6	1.18 (0.51–2.76)
*Clostridium* sp. infection, *n* (%)	3 (9.7%)	28 (10.1%)	0.94	0.95 (0.27–3.33)
Cranial traumatism, *n* (%)	3 (9.7%)	21 (7.6%)	0.6	1.3 (0.36–4.65)
Cardiovascular disease, *n* (%)	19 (61.3%)	176 (63.5%)	0.8	0.9 (0.42–1.94)
Chronic heart failure, *n* (%)	6 (19.4%)	97 (35%)	0.1	0.44 (0.17–1.12)
Coronary disease, *n* (%)	6 (19.4%)	70 (25.3%)	0.66	0.71 (0.28–1.8)
Myocardial infarction, *n* (%)	3 (9.7%)	30 (10.8%)	1	0.88 (0.25–3.07)
Arterial peripheral disease, *n* (%)	4 (12.9%)	24 (8.7%)	0.4	1.56 (0.5–4.87)
Intracranial haemorrhage, *n* (%)	7 (22.6%)	37 (13.4%)	0.17	1.89 (0.76–4.07)
Cerebrovascular disease, *n* (%)	13 (41.9%)	109 (39.4%)	0.84	1.11 (0.52–2.36)
Chronic and obstructive respiratory disease, *n* (%)	7 (22.6%)	38 (13.7%)	0.18	1.83 (0.73–4.55)
Chronic kidney disease, *n* (%)	6 (19.4%)	64 (23.1%)	0.82	0.8 (0.31–2.03)
Diabetes mellitus, *n* (%)	9 (29%)	96 (34.7%)	0.53	0.71 (0.34–1.74)
Thyroid gland disorders, *n* (%)	3 (9.7%)	22 (7.9%)	0.73	1.2 (0.35–4.41)
GI disorders (ulcers, bleeding history), *n* (%)	2 (6.5%)	38 (13.7%)	0.39	0.43 (0.09–1.89)
Malignancies, *n* (%)	5 (16.1%)	58 (20.9%)	0.64	0.72 (0.26–1.97)
Localised malignancy, *n* (%)	4 (12.9%)	26 (9.4%)	0.52	1.43 (0.46–4.4)
Metastases, *n* (%)	2 (6.5%)	33 (11.9%)	0.55	0.51 (0.11–2.23)
Immunosuppression, *n* (%)	12 (38.7%)	92 (33.2%)	0.53	1.27 (0.59–2.72)
Prior corticosteroid therapy, *n* (%)	8 (25.8%)	70 (25.3%)	1	1.02 (0.44–2.4)
Neurocognitive disorders, *n* (%)	5 (16.1%)	80 (28.9%)	0.13	0.47 (0.17–1.27)
Chemotherapy, *n* (%)	2 (6.5%)	24 (8.7%)	1	0.72 (0.16–3.23)
Obesity, *n* (%)	16 (51.6%)	114 (41.2%)	0.26	1.52 (0.72–3.2)
Liver disease, *n* (%)	2 (6.5%)	37 (13.4%)	0.39	0.44 (0.1–1.95)
Use of vasopressors, *n* (%)	12 (38.7%)	119 (43%)	0.65	0.83 (0.39–1.79)
Use of corticosteroids, *n* (%)	17 (54.8%)	105 (37.9%)	0.08	1.98 (0.94–4.2)
Use of antihypertensives, *n* (%)	6 (19.4%)	52 (18.8%)	0.93	1.03 (0.4–2.66)
Viral pneumonia, *n* (%)	4 (12.9%)	67 (24.2%)	0.18	0.46 (0.15–1.37)
Bacterial pneumonia, *n* (%)	21 (67.7%)	154 (55.6%)	0.19	1.67 (0.76–3.69)
Type of carbapenemase (single MBL Ref)			0.25	
KPC, *n* (%)	7 (22.6%)	94 (33.9%)	0.12	0.41(0.13–1.26)
OXA48, *n* (%)	10 (32.3%)	61 (22%)	0.	0.91 (0.32–2.6)
OXA48/KPC + NDM, *n* (%)	7 (22.6%)	83 (30%)	0.07	0.47 (0.15–1.43)
MBL, *n* (%)	7 (15.2%)	39 (14.1%)		
Colonisation with CR Enterobacterales, *n* (%)	14 (45.2%)	140 (50.5%)	0.57	0.8 (0.38–1.69)
Unidentified source, *n* (%)	23 (74.2%)	172 (62.1%)	0.18	1.75 (0.75–4.06)
Respiratory, *n* (%)	2 (6.5%)	31 (11.2%)	0.19	0.54 (0.12–2.4)
Urinary, *n* (%)	2 (6.5%)	47 (17%)	0.19	0.33 (0.07–1.46)
Central line, *n* (%)	4 (12.9%)	20 (7.2%)	0.28	1.9 (0.6–5.97)
Others, *n* (%)	2 (1.9%)	5 (2.5%)	0.5	0.62 (0.11–3.3)
Polymicrobial BSI, *n* (%)	9 (29%)	62 (22.4%)	0.4	1.41 (0.62–3.23)
*Candida* sp. co-isolation, *n* (%)	1 (3.2%)	12 (4.3%)	1	0.73 (0.09–5.86)
*Acinetobacter baumannii* co-isolation, *n* (%)	3 (9.7%)	17 (6.1%)	0.43	1.63 (0.45–5.94)
Exposure				
Carbapenems, *n* (%)	13 (41.9%)	120 (43.3%)	0.88	0.94 (0.44–2)
Colistin, *n* (%)	8 (25.8%)	68 (24.5%)	0.87	1.06 (0.45–2.05)
Cephalosporins, *n* (%)	12 (38.7%)	97 (35%)	0.68	1.17 (0.54–2.51)
Aminoglycosides, *n* (%)	0 (0%)	10 (3.6%)	0.6	0.81 (0.2–3.2)
Clindamycin, *n* (%)	4 (12.9%)	17 (6.1%)	0.24	2.26 (0.71–7.22)
Tigecycline, *n* (%)	1 (3.2%)	11 (4%)	1	0.8 (0.1–6.46)
Piperacillin/tazobactam, *n* (%)	5 (16.1%)	72 (26.2%)	0.22	0.54 (0.2–1.46)
Linezolid, *n* (%)	2 (6.5%)	54 (19.5%)	0.07	0.28 (0.06–1.23)
Fluoroquinolones, *n* (%)	4 (12.9%)	58 (20.9%)	0.29	0.55 (0.18–1.55)
Metronidazole, *n* (%)	3 (9.7%)	35 (12.6%)	0.78	0.74 (0.21–2.56)
Penicillins, *n* (%)	0 (0%)	22 (8%)	0.14	
Inappropriate initial therapy, *n* (%)	25 (80.6%)	209 (75.5%)	0.52	1.35 (0.53–3.44)
Initial therapy				
Fluoroquinolones, *n* (%)	3 (9.7%)	18 (6.5%)	0.45	1.54 (0.42–5.56)
Tigecycline, *n* (%)	1 (3.2%)	19 (6.9%)	0.7	0.45 (0.05–3.5)
Aminoglycosides, *n* (%)	0%	6 (100%)	1	
Initial therapy group				
C/AVI (+/−AZT), *n* (%)	1 (3.2%)	31 (11.2%)	0.33	
Colistin, *n* (%)	12 (38.7%)	86 (31%)		
Other therapies, *n* (%)	18 (58.1%)	160 (57.8%)		
Definitive				
C/AVI (+/−AZT), *n* (%)	8 (25.8%)	90 (32.5%)	0.3	
Colistin, *n* (%)	17 (54.8%)	112 (40.4%)		
Other therapies, *n* (%)	6 (19.4%)	75 (27.1%)		
Laboratory results				
Urea (mg/dL), median (IQR), *N* = 303	51 (37.25–91)	67 (38–109)	0.558	
Procalcitonin (ng/mL), median (IQR), *N* = 298	3.89 (0.81–24.95)	3.72 (0.745–18.64)	0.782	
Creatinine (mg/dL), median (IQR), *N* = 301	0.9 (0.545–1.9)	0.9 (0.53–1.7)	0.94	
C Reactive Protein (mg/L), median (IQR), *N* = 298	169.7 (82.1–253.7)	157.7 (74.3–241.1)	0.556	
D-Dimers (ng/mL), median (IQR), *N* = 204	1757 (733–3927)	1240 (752–2861)	0.777	
Thrombocytes (10^3^/μL), median (IQR), *N* = 302	149 (108–240)	200 (118–266)	0.972	
AST (U/L), median (IQR), *N* = 288	40 (29–55)	48 (33–82)	0.959	
ALT (U/L), median (IQR), *N* = 288	18 (14.75–47.5)	35 (20–81)	0.535	
Neutrophils (10^3^/μL), median (IQR)	14.17 (9.62–21.4)	10.98 (7.2–18.85)	0.986	
Leukocytes (10^3^/μL), median (IQR)	15.29 (10.49–22.36)	12.84 (8.65–19.59)	0.921	
Monocytes (10^3^/μL), median (IQR)	0.62 (0.29–0.85)	0.52 (0.26–0.84)	0.766	
Erythrocytes (10^6^/μL), median (IQR)	3.35 (3.01–3.8)	3.3 (2.76–3.73)	0.434	
Lymphocytes (10^3^/μL), median (IQR)	0.44 (0.33–0.79)	0.64 (0.41–1.29)	0.713	
Fibrinogen (mg/dL), median (IQR), *N* = 284	404 (284–550)	427 (336–528)	0.209	
Haemoglobin (g/dL), median (IQR)	10.3 (9.05–11.75)	9.3 (7.8–10.9)	0.119	
Sodium (meq/mol), median (IQR)	142 (138.5–150)	141 (138–146)	0.248	
Potassium (meq/mol), median (IQR)	4.3 (3.6–4.5)	4 (3.5–4.4)	0.511	
Glucose (mg/dL), median (IQR), *N* = 278	128 (91.5–222)	119 (93–156)	0.298	
Albumin (g/dL), median (IQR), *N* = 217	2.8 (2.45–3)	2.72 (2.3–3)	0.924	

* *p* < 0.05; ALT: Alanine Aminotransferase; AST: Aspartate Aminotransferase; AZT: aztreonam; BSI: bloodstream infection; C/AVI: ceftazidime/avibactam CI95%: confidence interval 95%; ICU: intensive care unit; IQR: interquartile range; CR: carbapenem-resistant; GI: gastrointestinal; MBL: metallo beta lactamase; OR: odds ratio.

**Table 5:** Multivariate analysis of factors associated withrecurrent or persistent bacteraemia.

Feature	B	S.E.	WALD	*p*	aOR (CI95%)
Time from admission to BSI	−0.054	0.022	6.128	0.013 *	0.94 (0.9–0.98)
Source of bacteraemia (No source Ref.)			5.55	0.13	
Catheter	0.76	0.63	1.461	0.22	2.14 (0.62–7.37)
Respiratory	−0.32	0.788	0.173	0.67	0.72 (0.15–3.37)
Urinary, biliary, skin	−1.43	0.779	3.373	0.06	0.23 (0.05–1.1)
Type of carbapenemase single OXA48 or MBL vs. ref double MBL or KPC)	0.903	0.402	5.048	0.025 *	2.46 (1.12–5.41)
Prior linezolid	−1.039	0.767	1.834	0.17	0.35 (0.07–1.59)
Corticosteroids	0.586	0.403	2.117	0.14	1.79 (0.81–3.95)
Constant	−1.54	0.454	16.704	0	0.15

* *p* < 0.05; aOR: adjusted odds ratio; AZT: aztreonam; BSI: bloodstream infection; CI95%: confidence interval 95%; MBL: metallo beta lactamase; REF: Reference.

**Table 6:** Cox proportional-hazards model with initial therapy for 30-day mortality.

	Beta	SE	WALD	*p*	aHR	CI95%
Age	0.136	0.011	157.216	0.000 *	1.14	1.12–1.17
Age × lnTime	−0.063	0.004	228.566	0.000 *	1.14	0.93–0.95
Charlson score	0.033	0.911	0.911	0.34	1.03	0.96–1.10
Inadequate initial therapy	0.185	0.214	0.75	0.38	1.20	0.79–1.83
Vasopressor use	0.436	0.16	7.249	0.007 *	1.54	1.12–2.12
Corticotherapy	0.238	0.155	2.343	0.126	1.26	0.94–1.72
Viral pneumonia	−0.035	0.182	0.037	0.848	0.966	0.67–1.38
Cranial traumatism	−0.662	0.346	3.65	0.056	0.51	0.26–1.01
ICU admission	0.191	0.203	0.883	0.347	1.21	0.81–1.8
MBL (vs. non MBL)	−0.073	0.159	0.212	0.645	0.93	0.68–1.26
*Acinetobacter* sp. co-isolation	0.452	0.273	2.753	0.097	1.57	0.92–2.68
Source of BSI (No source REF)			8.778	0.067		
Catheter	−0.232	0.332	0.490	0.484	0.793	0.41–1.51
Respiratory	0.216	0.237	0.833	0.361	1.241	0.78–1.97
Urinary	−0.498	0.259	3.705	0.054	0.608	0.36–1.09
Other	0.720	0.437	2.712	0.1	2.05	0.87–4.84
Initial treatment (Colistin REF)			2.917	0.233		
C/AVI(+/−AZT)	−0.481	0.327	2.156	0.14	0.61	0.32–1.17
Other therapies	−0.18	0.185	1.045	0.30	0.82	0.57–1.18

* *p* < 0.05; aHR: adjusted hazards ratio; AZT: aztreonam; C/AVI: ceftazidime/avibactam;CI95%: confidence interval 95%; ICU: intensive care unit; MBL: metallo beta lactamase; REF: Reference.

**Table 7:** Cox proportional-hazards model in landmark approach with definitive therapy for 30-day mortality.

Feature	Beta	SE	WALD	*p*	aHR	CI95%
Age	0.129	0.013	101.244	0.000 *	1.14	1.11–1.16
Age × lnTime	−0.06	0.005	154.733	0.000 *	0.942	0.93–0.95
Charlson score	0.026	0.043	0.375	0.54	1.02	0.94–1.11
Inadequate initial therapy	0.264	0.227	1.338	0.189	1.34	0.86–2.1
Vasopressor use	0.249	0.2	1.56	0.212	1.28	0.86–1.89
Corticotherapy	0.338	0.186	3.319	0.06	1.4	0.97–2.01
Viral pneumonia	−0.03	0.225	0.018	0.892	0.97	0.62–1.5
Cranial traumatism	−0.594	0.38	2.437	0.11	0.55	0.26–1.16
ICU admission	0.299	0.227	1.728	0.189	1.34	0.86–2.1
MBL (vs. non MBL)	−0.056	0.193	0.085	0.77	0.94	0.64–1.38
*Acinetobacter* sp. co-isolation	0.223	0.272	4.14	0.52	1.24	0.73–2.13
Source of BSI (No source REF)			8.329	0.08		
Central line	−0.61	0.387	2.493	0.11	0.54	0.25–1.15
Respiratory	0.16	0.275	0.37	0.54	1.18	0.69–2.02
Urinary	−0.332	0.294	1.274	0.25	0.71	0.4–1.27
Other	0.96	0.495	3.767	0.052	2.18	0.99–6.88
Definitive treatment (Colistin REF)			7.87	0.02 *		
C/AVI(+/−AZT)	−0.142	0.198	3.553	0.48	0.86	0.58–1.28
Other therapies	−0.761	0.272	7.835	0.005 *	0.46	0.27–0.80

* *p* < 0.05; aHR: adjusted hazards ratio; AZT: aztreonam; C/AVI: ceftazidime/avibactam; BSI:bloodstream infection;CI95%: confidence interval 95%; ICU: intensive care unit; MBL: metallo beta lactamase; REF: Reference.

**Table 8:** Inverse propensity score weightinglogistic regression for 30-day mortality after applying the weight function in the main cohort of patients receiving colistin or ceftazidime/avibactam+/−aztreonam (*N* = 202).

Variable	B	S.E.	WALD	*p*	Exp(B)	CI95%
C/AVI (+/−AZT) vs. Colistin (REF)	−0.717	0.326	4.826	0.028	0.488	0.258–0.926
Constant	1.399	0.233	36.026	0.000	4.053	

AZT: aztreonam; C/AVI: ceftazidime/avibactam; REF: reference.

**Table 9:** Inverse propensity score weightinglogistic regression for 30-day mortality after applying the weight function in the group of patients infected with OXA48/KPC/OXA48 + KPC Enterobacterales receiving colistin or ceftazidime/avibactam: Sensitivity analysis (*N* = 110).

Variable	B	S.E.	WALD	*p*	Exp(B)	CI95%
C/AVI (+/−AZT) vs. Colistin (REF)	−0.695	0.446	2.424	0.12	0.499	0.208–1.197
Constant	1.362	0.297	21.044	0.000	3.903	

AZT: aztreonam; C/AVI: ceftazidime/avibactam; REF: reference.

## Data Availability

The datasets generated and analysed during the current study are not publicly available because they contain individual patient-level clinical data collected under a waiver of informed consent for retrospective analysis, but they are available from the corresponding author on reasonable request.

## Supplementary Materials

The following supporting information can be downloaded at: https://www.mdpi.com/article/10.3390/microorganisms14092102/s1, Table S1: Univariate analysis of 30-day mortality; Table S2: Univariate analysis of 7-day mortality; Table S3: Multivariable logistic regression for 7-day mortality; Table S4: Univariate analysis of 14-day mortality; Table S5: Multivariable logistic regression for 14-day mortality; Table S6: Carbapenemase class, definitive therapy and 30-day mortality by calendar epoch; Table S7: Phenotypic ceftazidime/avibactam susceptibility, composition of the definitive regimen and timing of ceftazidime/avibactam initiation; Table S8: Weighted analysis adjusted for and stratified by calendar period; Table S9: Weighted analysis stratified by carbapenemase class; Table S10: Comparison of patients with and without a documented day-30 status and extremecase sensitivity analyses.
