# Supplementary material for: Ceftazidime–Avibactam Versus Colistin-Based Regimens for Carbapenem-Resistant Enterobacterales Bloodstream Infections: A Six-Year Retrospective Cohort and 30-Day Mortality Analysis
Source: Microorganisms. 2026 Sep 19;14(9):2102. doi: 10.3390/microorganisms14092102 (PMC13609087; doi:10.3390/microorganisms14092102)
Supplement: Supplementary file 1 [file microorganisms-14-02102-s001.zip › microorganisms-4524557-supplementary.pdf]

microorganisms

Supplementary Materials

Ceftazidime–Avibactam versus Colistin-Based Regimens for Carbapenem-Resistant Enterobacterales Bloodstream Infections: A Six-Year Retrospective Cohort and 30-Day Mortality Analysis

Victor-Pierre Ormeneanu, Anca Zanfirescu, Corina Andrei, Florin Dumitru, Flavius Anghel, Răzvan Adam and Simona Negreș

Table S1. Univariate analysis of demographic, clinical and laboratory variables in relation to 30 day mortality in patients with documented status in bloodstream infections caused by Carbapenemase producing Enterobacterales (N=275)

Variable	Dead (N=198)	Alive (N=77)	P	OR CI95%
Age, median (IQR)	69.5 [60-78]	65 [53-74]	0.07	1.63 (0.95-2.77)
Charlson Comorbidity Index, median (IQR)	6 [4-8]	5 [3-7]	0.045*	1.58 (0.9 -2.68)
Male sex, n (%)	111 (56.1%)	48 (62.3%)	0.34	0.77 (0.44-1.32)
Length of stay, median (IQR)	24 [13-35]	45 [24-66]	<0.001*	
Number of days until bacteraemia, median (IQR)	14 (8-24)	13 [7-24]	0.3	
ICU Length of stay, median (IQR)	11 [6-23]	15 [0-37]	0.45	
History of smoking, n (%)	41 (20.7%)	22 (28.6%)	0.16	0.65 (0.35-1.19)
Active smoker, n (%)	23 (11.6%)	16 (20.8%)	0.051	0.5 (0.24-1.01)
Chronic alcohol consumption, n (%)	32 (16.2%)	13 (16.9%)	0.88	0.94 (0.46-1.92)
Recent surgical interventions, n (%)	69 (34.8%)	36 (46.8%)	0.06	0.608 (0.35-1.04)
ICU stay, n (%)	157 (79.3%)	50 (64.9%)	0.01*	2.07 (1.16-3.7)
Clostridium infection, n (%)	19 (9.3%)	9 (11.7%)	0.55	0.77 (0.33-1.8)
Cranial traumatism, n (%)	11 (5.6%)	10 (13%)	0.04*	0.39 (0.16-0.97)
Cardiovascular disease, n (%)	129 (65.2%)	45 (58.4%)	0.3	1.33 (0.77-2.28)
Chronic heart failure, n (%)	70 (35.4%)	21 (27.3%)	0.2	1.46 (0.81-2.6)
Coronary disease, n (%)	49 (24.7%)	19 (23.4%)	0.81	1.08 (0.5-2)

Myocardial infarction, n (%)	19 (9.6%)	10 (13%)	0.41	0.71 (0.31-1.61)
Arterial peripheral disease, n (%)	18 (9.1%)	6 (7.8%)	0.81	1.18 (0.45-3.1)
Intracranial hemorrhage, n (%)	25 (12.6%)	16 (20.8%)	0.08	0.55 (0.27-1.1)
Cerebrovascular disease, n (%)	76 (38.4%)	34 (44.2%)	0.38	0.78 (0.46-1.34)
Chronic and obstructive respiratory disease, n (%)	26 (13.1%)	15 (19.5%)	0.18	0.62 (0.31-1.26)
Chronic kidney disease, n (%)	49 (24.7%)	14 (18.2%)	0.24	1.48 (0.76-2.87)
Diabetes mellitus, n (%)	69 (34.8%)	26 (33.8%)	0.8	1.05 (0.6-1.83)
Thyroid gland disorders, n (%)	13 (6.6%)	9 (11.7%)	0.2	0.53 (0.21-1.3)
GI disorders (ulcers. bleeding history), n (%)	25 (12.6%)	10 (13%)	0.93	0.96 (0.44-2.1)
Malignancies, n (%)	46 (23.2%)	14 (18.2%)	0.36	1.3 (0.69-2.65)
Localised malignancy, n (%)	20 (9.8%)	9 (11.7%)	0.66	0.84 (0.36-1.96)
Metastases, n (%)	26 (13.1%)	7 (9.1%)	0.35	1.51 (0.62-3.64)
Immunosuppression, n (%)	71 (35.9%)	26 (33.8%)	0.74	1.1 (0.63-1.91)
Prior corticosteroid therapy, n (%)	52 (26.3%)	20 (26%)	0.96	1.02 (0.55-1.85)
Neurocognitive disorders, n (%)	57 (28.8%)	15 (19.5%)	0.11	1.67 (0.87-3.18)
Chemotherapy, n (%)	19 (9.6%)	5 (6.5%)	0.41	1.53 (0.55-4.25)
Obesity, n (%)	86 (43.4%)	38 (49.4%)	0.37	0.78 (0.46-1.34)
Liver disease, n (%)	27 (13.6%)	9 (11.7%)	0.66	1.19 (0.53-2.67)
Use of vasopressors, n (%)	114 (57.6%)	12 (15.6%)	<0.001*	7.35 (3.73-14.5)
Use of corticosteroids, n (%)	86 (43.4%)	29 (37.7%)	0.38	1.27 (0.74-2.18)
Use of antihypertensives, n (%)	33 (16.7%)	19 (24.7%)	0.12	0.61 (0.32-1.16)
Viral pneumonia, n (%)	58 (29.3%)	7 (9.1%)	<0.001*	4.14 (1.8-9.55)
Bacterial pneumonia, n (%)	123 (62.1%)	37 (48.1%)	0.03*	1.77 (1.04-3.02)
Source of BSI			0.162	
Respiratory, n (%)	24 (12.1%)	5 (6.5%)		
Urinary, biliary, soft tissues, n (%)	28 (14.1%)	17 (22.1%)		
Central line, n (%)	11 (5.6%)	7 (9.1%)		
Unidentified source, n (%)-Ref	135 (68.2%)	48 (62.3%)		
Type of carbapenemase			0.5	

KPC, n (%)	69 (34.8%)	23 (29.9%)		
OXA48, n (%)	40 (20.2%)	17 (22.1%)		
OXA48/KPC+NDM, n (%)	61 (30.8%)	21 (27.3%)		
MBL, n (%)-Ref	28 (14.1%)	16 (20.8%)		
Colonisation with resistant CR Enterobacteriaceae, n (%)	96 (48.5%)	42 (54.5%)	0.36	0.78 (0.46-1.33)
Polymicrobial BSI, n (%)	48 (24.2%)	17 (22.1%)	0.7	1.13 (0.6-2.12)
Acinetobacter sp co-isolation, n (%)	16 (8.1%)	2 (2.6%)	0.11	3.3 (0.74-14.7)
Candida sp. co-isolation, n (%)	12 (6.3%)	1 (1.3%)	0.12	4.9 (0.62-38.4)
Exposure				
Carbapenems, n (%)	90 (45.5%)	28 (36.4%)	0.17	1.45 (0.84-2.5)
Colistin, n (%)	50 (25.3%)	21 (27.3%)	0.7	0.9 (0.49-1.63)
Cephalosporins, n (%)	69 (34.8%)	31 (40.3%)	0.4	0.79 (0.46-1.36)
Linezolid, n (%)	43 (21.7%)	9 (11.7%)	0.06	2.1 (0.96-4.54)
Metronidazol, n (%)	26 (13.1%)	8 (10.4%)	0.68	1.3 (0.56-3.02)
Clyndamicin, n (%)	10 (5.1%)	9 (11.7%)	0.06	0.4 (0.15-1.03)
Piperacillin, n (%)	57 (29.1%)	14 (18.2%)	0.06	1.85 (0.95-3.56)
Fluoroquinolones, n (%)	42 (21.2%)	14 (18.2%)	0.57	1.21 (0.61-2.37)
Aminopenicillins, n (%)	12 (6.1%)	8 (10.4%)	0.3	0.56 (0.22-1.43)
Inappropriate initial therapy, n (%)	156 (78.8%)	55 (71.4%)	0.19	1.49 (0.81-2.71)
Initial therapy				
C/AVI (+/-AZT), n (%)	13 (6.6%)	13 (16.9%)	0.01*	
Colistin, n (%)	73 (36.9%)	19 (35.4%)		
Others, n (%)	112 (56.6%)	45 (58.4%)		
Fluoroquinolones, n (%)	14 (7.1%)	5 (6.5%)	0.86	1.1 (0.38-3.15)
Tigecycline, n (%)	13 (6.6%)	5 (6.5%)	1	1.01 (0.34-2.94)
Definitive therapy				
C/AVI (+/-AZT) , n (%)	57 (28.8%)	29 (37.7%)	0.07	0.66 (0.38-1.16)
Colistin, n (%)	92 (46.5%)	24 (31.2%)		1.92 (1.1-3.35)
Others, n (%)	49 (24.7%)	24 (31.2%)		
		Laboratory results		
Parameter				
Urea (mg/dL), median (IQR), N=275	72.9 [46-116.75]	50 [31-75]	<0.001*	
Procalcitonine (ng/mL), median (IQR), N=271	5.76 [1.22-22.75]	1.12 [0.34-5.5]	<0.001*	
Creatinine (mg/dL), median (IQR), N=273	1.1 [0.7-2]	0.71 [0.5-1.11]	<0.001*	

C Reactive Protein (mg/L), median (IQR), N=271	169.45 [79.55-251.9]	151.05 {70.12-221.57]	0.113
D-Dimers (ng/mL), median (IQR), N=180	1770 [853-3276]	839 [555.7-2272]	0.003*
Thrombocytes (10^3/uL), median (IQR)N=274	173.5 [113-258.5]	213 [154.25-262.5]	0.02*
AST (U/L), median (IQR), N=260	48 [30.7-85.5]	36 [24.25-62]	0.01*
ALT (U/L), median (IQR), N=260	28.5 [17-61.2]	37 [20-90.7]	0.1
Neutrophils.(10^3/uL), median (IQR) N=273	12.28 [7.43-18.5]	8.77 [7.2-13.8]	0.018*
Leucocytes (10^3/uL), median (IQR), N=273	13.73 [8.6-19.57]	11.45 [8.65-15.98]	0.056
Monocytes.(10^3/uL), median (IQR) N=273	0.44 [0.22-0.76]	0.62 [0.35-0.86]	0.059
Erythrocytes (10^6/uL), median (IQR), N=275	3.11 [2.64-3.57]	3.38 [2.94-3.85]	0.004*
Lymphocytes (10^3/uL), median (IQR), N=273	0.71[0.4-1.17]	0.94 [0.6-1.4]	0.007*
Fibrinogen (mg/dL), median, (IQR) N=255	427 [322-516.5]	487.5 [386.75-557.75]	0.03*
Hemoglobine (g/dL), median (IQR), N=273	8.9 [7.72-10.4]	9.7 [8.7-11.3]	<0.001*
Sodium (meq/mol), median, (IQR) N=275	140 [136-145.75]	139 [135-143]	0.17
Potassium (meq/mol), median (IQR), N=275	3.9 [3.4-4.3]	3.7 [3.5-4]	0.12
Glucose (mg/dL), median (IQR), N=272	110 [85-158]	115 [93-139]	0.6
Albumin (g/dL), median (IQR), N=200	2.6 [2.2-2.9]	2.7 [2.4-3.1]	0.01 *

*p<0.05

AZT: Aztreonam; ALT: Alanine Aminotransferase; AST: Aspartate Aminotransferase; BSI: bloodstream infection; GI: gastrointestinal; ICU: Intensive Care Unit; IQR: Interquartilic range; C/AVI: ceftazidime/avibactam; CI95%: confidence interval 95%; CR: carbapenem-resistant; MBL: metallo beta lactamase; OR: odds ratio

Table S2. Univariate analysis of demographic, clinical and laboratory variables in relation to 7 day mortality in patients with documented status with bloodstream infections caused by Carbapenemase producing Enterobacterales (N=303)

Feature	Dead (N=104)	Alive (N=199)	p	OR CI95%
Age, median (IQR)	67.5 [57-76]	69 [58-77]	0.81	
Charlson Comorbidity Index, median (IQR)	6 [4-8]	6 [4-8]	0.74	
Male sex,n (%)	62 (60.2%)	112 (56%)	0.48	1.19 (0.73-1.93)
Lenghth of stay, median (IQR)	23.5 [13-35]	46 [26-66]	<0.001*	
Number of days until bacteraemia, median (IQR)	14 [8-24]	14 [8-26]	0.84	
ICU length of stay, median (IQR)	10 [6-23]	14.5 [2-37]	0.056	
History of smoking, n (%)	19 (18.5%)	49 (24.6%)	0.2	0.68 (0.37-1.24)
Active smoker, n (%)	11 (10.6%)	30 (15.1%)	0.27	0.66 (0.31-1.39)
Chronic alcohol consumption, n (%)	18 (17.3%)	30 (15.1%)	0.61	1.18 (0.62-2.23)
Recent surgical interventions, n (%)	33 (31.7%)	79 (39.7%)	0.17	0.7 (0.42-1.17)
ICU stay, n (%)	85 (81.7%)	132 (66.3%)	0.005*	2.27 (1.27-4.05)
Clostridium infection, n (%)	10 (9.6%)	21 (10.6%)	0.84	0.9 (0.4-2)
Cranial traumatism	6 (5.8%)	18 (9%)	0.37	0.61 (0.23-1.6)
Cardiovascular disease	67 (64.4%)	125 (62.8%)	0.78	1.07 (0.65-1.76)
Chronic heart failure, n (%)	35 (33.7%)	67 (33.7%)	0.99	1.02 (0.6-1.65)
Coronary disease, n (%)	25 (24%)	50 (25.1%)	0.83	0.94 (0.54-1.64)
Myocardial infarction, n (%)	10 (9.6%)	23 (11.6%)	0.6	0.81 (0.37-1.78)
Arterial peripheral disease, n (%)	6 (5.8%)	22 (11%)	0.2	0.5 (0.19-1.28)
Intracranial hemorrhage, n (%)	11 (10.6%)	32 (16.1%)	0.22	0.61 (0.29-1.28)
Cerebrovascular disease, n (%)	31 (29.8%)	88 (44.2%)	0.015*	0.53 (0.32-0.88)
Chronic and obstructive respiratory disease, n (%)	12 (11.5%)	32 (16.1%)	0.3	0.68 (0.33-1.39)
Chronic kidney disease, n (%)	30 (28.8%)	40 (20.1%)	0.08	1.61 (0.93-2.79)
Diabetes mellitus, n (%)	42 (40.4%)	61 (30.7%)	0.09	1.53 (0.93-2.51)
Thyroid gland disorders, n (%)	8 (7.7%)	17 (8.5%)	1	0.9 (0.37-2.14)
GI disorders (ulcers. bleeding history), n (%)	15 (14.4%)	25 (12.6%)	0.65	1.17 (0.58-2.34)

Malignancies, n (%)	19 (18.3%)	43 (21.6%)	0.55	0.81 (0.44-1.48)
Localised malignancy, n (%)	6 (5.8%)	24 (12.1%)	0.1	0.44 (0.17-1.13)
Metastases, n (%)	14 (13.5%)	20 (10.1%)	0.44	1.39 (0.67-2.88)
Immunosuppression, n (%)	40 (38.5%)	62 (31.2%)	0.2	1.38 (0.84-2.27)
Prior corticosteroid therapy, n (%)	29 (27.9%)	47 (23.6%)	0.4	1.25 (0.72-2.14)
Neurocognitive disorders, n (%)	28 (26.9%)	55 (27.6%)	0.89	0.96 (0.56-1.64)
Chemotherapy, n (%)	14 (13.5%)	11 (5.5%)	0.02*	2.66 (1.16-6.09)
Obesity, n (%)	43 (41.3%)	85 (42.7%)	0.81	0.94 (0.58-1.53)
Liver disease, n (%)	17 (16.3%)	21 (10.6%)	0.2	1.66 (0.83-3.3)
Use of vasopressors, n (%)	71 (68.3%)	58 (29.1%)	<0.001*	5.23 (3.13-8.74)
Use of corticosteroids, n (%)	43 (41.3%)	76 (38.2%)	0.52	1.14 (0.7-1.85)
Use of antihypertensives, n (%)	11 (10.6%)	46 (23.1%)	0.008*	0.39 (0.19-0.79)
Viral pneumonia, n (%)	34 (32.7%)	36 (18.1%)	0.004*	2.2 (1.27-3.8)
Bacterial pneumonia, n (%)	58 (55.8%)	115 (57.8%)	0.73	0.92 (0.57-1.49)
Type of carbapenemase (single MBL as Ref)			0.136	
KPC, n (%)	38 (36.5%)	62 (31.2%%)	0.382	0.73(0.36-1.47)
OXA48, n (%)	18 (17.3%)	49 (24.6%)	0.04*	0.43 (0.19-0.96)
OXA48/KPC+NDM, n (%)	27 (26%)	63 (31.7%)	0.07	0.51 (0.25-1.06)
MBL, n (%)	21 (20.2%)	25 (12.6%)		
Colonisation with resistant CR Enterobacteriaceae, n (%)	49 (47.1%)	102 (51.3%)	0.49	0.84 (0.52-1.36)
Source of BSI (No source REF)			0.18	
Respiratory, n (%)	11 (11.1%)	21 (10.6%)	0.5	0.78 (0.36-1.71)
Urinary, n (%)	12 (11.5%)	35 (17.6%)	0.09	0.53 (0.26-1)
Central line, n (%)	4 (3.8%)	19 (9.5%)	0.052	0.33 (0.1-1.01)
Others, n (%)	2 (1.9%)	5 (2.5%)	0.5	0.62 (0.11-3.3)
Polymicrobial BSI, n (%)	20 (19.2%)	50 (25.1%)	0.31	0.71 (0.4-1.27)
Candida sp. co-isolation, n (%)	6 (5.8%)	7 (3.5%)	0.38	1.68 (0.54-5.13)
Acinetobacter baumannii co-isolation, n (%)	9 (8.7%)	10 (5%)	0.21	1.79 (0.7-4.55)
Exposure				
Carbapenems, n (%)	48 (46.2%)	82 (41.2%)	0.4	1.22 (0.75-1.97)
Colistin, n (%)	32 (30.8%)	44 (22.2%)	0.1	1.55 (0.91-2.65)
Cephalosporins, n (%)	33 (31.7%)	75 (37.7%)	0.3	0.76 (0.46-1.27)

Aminoglycosides, n (%)	3 (2.9%)	7 (3.5%)	0.77	0.81 (0.2-3.2)
Clindamycine, n (%)	5 (4.8%)	16 (8%)	0.29	0.57 (0.2-1.6)
Tigecycline, n (%)	3 (4.5%)	9 (2.9%)	0.49	0.62 (0.16-2.36)
Piperacillin/tazobactam, n (%)	32 (31.1%)	44 (22.2%)	0.09	1.57 (0.92-2.69)
Linezolid, n (%)	22 (21.2%)	32 (16.1%)	0.27	1.4 (0.76-2.56)
Fluoroquinolones, n (%)	19 (18.3%)	42 (21.1%)	0.55	0.83 (0.45-1.52)
Metronidazole, n (%)	7 (6.7%)	31 (15.6%)	0.03*	0.39 (0.16-0.92)
Aminopenicillins, n (%)	8 (7%)	14 (7.8%)	0.8	1.12 (0.45-2.77)
Inappropriate initial therapy, n (%)	79 (76%)	151 (75.9%)	0.98	1 (0.57-1.75)
Initial therapy				
C/AVI(+/AZT), n (%)	8 (7.8%)	24 (12%)	0.08	0.44 (0.18-1.09)
Colistin, n (%)	41 (39.4%)	55 (27.6%)		1.7 (1.03-2.81)
Others, n (%)	54 (52.4%)	121 (60.5%)		0.61 (0.36-1.02
Fluoroquinolones	7 (6.7%)	14 (7%)	1	0.95 (0.37-2.44)
Tigecycline	9 (8.7%)	11 (5.5%)	0.33	1.62 (0.64-4.04)
Definitive therapy				
C/AVI(+/AZT), n (%)	22 (21.2%)	76 (38.2%)	0.006*	
Colistin, n (%)	47 (45.2%)	80 (40.2%)		
Others, n (%)	35 (33.7%)	43 (21.6%)		
		Laboratory results		
Urea (mg/dL), median (IQR), N=303	78 (46.48-120.5)	58 (36-96)	0.004*	
Procalcitonine (ng/mL), median (IQR), N=298	6.78 (1.13-28.5)	3.19 (0.61-13)	0.016*	
Creatinine (mg/dL), median (IQR), N=301	1.3 (0.7-2.14)	0.8 (0.5-1.5)	<0.001	
C Reactive Protein (mg/L), median (IQR), N=298	198 (80-280)	142.15 (74.25-217.15)	0.01*	
D-Dimers (ng/mL), median (IQR), N=204	2255 (896-3821)	1150 (713.5-2284)	0.01*	
Thrombocytes (10^3/uL), median (IQR), N=302	172 (104.5-252.5)	203 (138-271)	0.04*	
AST (U/L), median (IQR), N=288	55 (36-104)	37 (25.5-63)	<0.001*	
ALT (U/L), median (IQR), N=288	33 (19-72)	29 (16-61)	0.41	
Neutrophils (10^3/uL), median (IQR)	13.61 (9.02-21.04)	10.08 (6.86-15.37)	0.002	
Leucocytes (10^3/uL), median (IQR)	14.57 (9.61-22.21)	11.66 (8.38-17.06)	0.004*	

Monocytes (10^3/uL), median (IQR)	0.42 (0.22-0.77)	0.56 (0.26-0.84)	0.27	
Erythrocytes (10^6/uL), median (IQR)	3.14 (2.78-3.55)	3.22 (2.73-3.08)	0.38	
Lymphocytes (10^3/uL), median (IQR)	0.62 (0.39-0.99)	0.88 (0.53-1.33)	<0.001*	
Fibrinogen (mg/dL), median (IQR), N=284	444 (327-516.5)	435 (382-555.5)	0.746	
Hemoglobine (g/dL), median (IQR)	8.9 (7.8-10.2)	9.45 (8-10.9)	0.058	
Sodium (meq/mol), median (IQR)	140 (136-145.5)	140 (135-144)	0.19	
Potassium (meq/mol), median (IQR)	4 (3.5-4.5)	3.75 (3.37-4.2)	0.01*	
Glucose (mg/dL), median (IQR), N=278	113 (87.5-175.5)	110 (88-110)	0.27	
Albumin (g/dL), median (IQR), N=214	2.6 (2.1-2.9)	2.6 (2.28-2.9)	0.55	

*p<0.05

ALT: Alanine Aminotransferase; AST: Aspartate Aminotransferase;AZT: Aztreonam; BSI: bloodstream infecion; C/AVI: ceftazidime/avibactam; CI95%: confidence interval 95%; CR: carbapenem-resistant; GI: gastroinetestinal; ICU: Intensive Care Unit; IQR: Interquartilic range; MBL: metallo beta lactamase; OR: odds ratio; REF: reference

Table S3. Table 11Multivariate analysis of factors associated to 7 day mortality in bloodstream infections caused by Carbapenemase producing Enterobacterales

Variable	B	S.E.	WALD	P	aOR (CI95%)
Age	0.002	0.014	0.019	0.88	1.00 (0.97-1.02)
Charlson Comorbidity Index	0.02	0.07	0.084	0.77	1.02 (0.88-1.17)
Chemotherapy	1.41	0.58	5.79	0.01*	4.1 (1.3-12.93)
Viral pneumonia	0.502	0.366	1.88	0.171	1.65 (0.8-3.38)
Cerebrovascular disease	-0.401	0.339	1.405	0.23	0.66 (0.34-1.3)
Vasopressor necessity	1.859	0.338	30.25	0.000*	6.41 (3.3-12.44)
Antihypertensive medication	0.27	0.44	0.387	0.53	1.31 (0.55-3.15)
Source of BSI (NO source Ref.)			3.707	0.44	
Catether	-1.161	0.67	2.95	0.08	0.31 (0.08-1.17)
Respiratory	-0.301	0.46	0.413	0.52	0.74 (0.29-1.85)
Urinary	-0.37	0.444	0.714	0.39	0.68 (0.28-1.64)
Other	-0.53	0.98	0.299	0.58	0.58 (0.08-4.01)
Inadequate initial therapy	-0.161	0.353	0.208	0.64	0.85 (0.42-1.69)
Type of carbapenemase (single MBL REF)			4.36	0.22	
KPC	-0.414	0.44	0.867	0.352	0.66 (0.27-1.58)
OXA48	-0.934	0.47	3.95	0.047*	0.39 (0.15-0.98)

OXA48+NDM	-0.735	0.45	2.61	0.106	0.47 (0.21-1.26)
Prior metronidazole	-1.14	0.51	4.9	0.02*	0.31 (0.11-0.87)
Definitive therapy (Colistin therapy REF)					
C/AVI(+/-AZT)	-0.803	0.355	5.1	0.02*	0.44 (0.22-0.89)
Others	0.53	0.36	2.14	0.14	1.7 (0.83-3.4)
Constant	-0.942	0.848	1.232	0.267	0.39

*p<0.05

aOR: adjusted Odds Ratio; AZT: Aztreonam; BSI: bloodstream infection; C/AVI: ceftazidime/avibactam;CI95%: confidence interval 95%; MBL: metallo beta lactamase; REF: Reference

Table S4. Univariate analysis of demographic, clinical and laboratory variables in relation to 14 day mortality in patients with documented status with bloodstream infections caused by Carbapenemase producing Enterobacterales (N=297)

Feature	Dead (N=162)	Alive (=135)	P	OR CI95%
Age, median (IQR)	68 [59-77]	68.5 [56.75-76]	0.5	1.16 (0.73-1.84)
ICC, median (IQR)	6 [4-8]	6 [4-8]	0.68	1.01 (0.69-1.74)
Male sex, n (%)	94 (57.7%)	77 (57.5%)	0.97	1.01 (0.63-1.6)
Length of stay, median (IQR)	20.5 [11-32]	40 [26-59]	<0.001*	
Number of days until bacteraemia, median (IQR)	14 [7-24]	14 [8-25]	1	
ICU length of stay,median (IQR)	11 [6-21]	19 [2.5-34]	0.101	
History of smoking, n (%)	34 (20.9%)	33 (24.6%)	0.43	0.8 (0.46-1.39)
Active smoker, n (%)	18 (11%)	22 (16.4%)	0.23	0.63 (0.32-1.23)
Chronic alcohol consumption, n (%)	30 (18.4%)	18 (13.4%)	0.24	1.45 (0.77-2.74)
Recent surgical interventions, n (%)	61 (37.4%)	50 (37.3%)	0.98	1 (0.62-1.61)
ICU stay, n (%)	131 (80.4%)	84 (61.9%)	<0.001*	2.52 (1.49-4.23)
Clostridium infection, n (%)	14 (16.9%)	17 (14.1%)	0.341	0.65 (0.311-1.39)
Cranial traumatism, n (%)	10 (6.1%)	13 (9.7%)	0.28	0.6 (0.25-1.44)
Cardiovascular disease, n (%)	106 (65%)	83 (61.9%)	0.58	1.14 (0.71-1.8)
Chronic heart failure, n (%)	56 (34.4%)	44 (32.8%)	0.78	1.07 (0.66-1.74)
Coronary disease, n (%)	38 (23.3%)	35 (26.1%)	0.57	0.86 (0.5-1.46)
Myocardial infarction, n (%)	16 (9.8%)	16 (11.9%)	0.55	0.8 (0.38-1.67)
Arterial peripheral disease, n (%)	14 (8.6%)	13 (9.7%)	0.87	0.89 (0.39-1.93)
Intracranial hemorrhage, n (%)	22 (13.5%)	21 (15.7%)	0.59	0.84 (0.44-1.6)
Cerebrovascular disease, n (%)	64 (39.3%)	54 (40.3%)	0.9	0.95 (0.6-1.53)

Chronic and obstructive respiratory disease, n (%)	20 (12.3%)	24 (17.9%)	0.19	0.64 (0.33-1.22)
Chronic kidney disease, n (%)	41 (25.2%)	28 (20.9%)	0.37	1.27 (0.73-2.2)
Diabetes mellitus, n (%)	61 (37.4%)	41 (30.6%)	0.22	1.36 (0.83-2.2)
Thyroid gland disorders, n (%)	11 (6.7%)	14 (10.4%)	0.29	0.62 (0.27-1.42)
GI disorders (ulcers. bleeding history), n (%)	22 (13.5%)	18 (13.4%)	0.98	1.01 (0.51-1.96)
Malignancies, n (%)	34 (20.9%)	27 (20.1%)	0.88	1.04 (0.59-1.84)
Localised malignancy, n (%)	13 (8%)	16 (11.9%)	0.32	0.63 (0.29-1.38)
Metastases, n (%)	22 (13.5%)	12 (9%)	0.27	1.59 (0.75-3.34)
Immunosuppression, n (%)	60 (36.8%)	40 (29.9%)	0.2	1.37 (0.84-2.23)
Prior corticosteroid therapy, n (%)	43 (26.4%)	31 (23.1%)	0.52	1.19 (0.7-2.03)
Neurocognitive disorders, n (%)	41 (25.2%)	39 (29.1%)	0.51	0.81 (0.49-1.37)
Chemotherapy, n (%)	17 (10.4%)	8 (6%)	0.208	1.83 (0.76-4.39)
Obesity, n (%)	72 (44.2%)	54 (40.3%)	0.5	1.17 (0.73-1.86)
Liver disease, n (%)	24 (15.3%)	14 (9.7%)	0.16	1.69 (0.82-3.44)
Use of vasopressors, n (%)	101 (62%)	27 (20.1%)	<0.001*	6.46 (3.81-10.9)
Use of corticosteroids, n (%)	71 (43.6%)	45 (33.6%)	0.08	1.53 (0.95-2.45)
Use of antihypertensives, n (%)	26 (16%)	31 (23.1%)	0.11	0.63 (0.35-1.13)
Viral pneumonia, n (%)	50 (30.7%)	18 (13.4%)	<0.001*	2.85 (1.57-5.18)
Bacterial pneumonia, n (%)	100 (61.3%)	71 (53%)	0.14	1.41 (0.88-2.24)
Colonisation with resistant CR Enterobacteriaceae, n (%)	79 (48.5%)	71 (53%)	0.43	0.83 (0.52-1.32)
Type of carbapenemase (single MBL ref.)			0.88	
KPC, n (%)	55 (33.7%)	43 (32.1%)	0.77	0.9 (0.44-1.83)
OXA48, n (%)	33 (20.2%)	31 (23.1%)	0.45	0.74 (0.34-1.6)
OXA48/KPC+NDM, n (%)	48 (30.6%)	41 (29.4%)	0.59	0.82 (0.4-1.69)
MBL, n (%)	27 (16.6%)	19 (14.2%)		
Source of BSI (No source ref.)			0.06	
Respiratory, n (%)	16 (9.8%)	15 (11.2%)	0.25	0.64 (0.3-1.37)
Urinary, n (%)	19 (11.7%)	26 (19.4%)	0.02*	0.47 (0.24-0.91)
Central line, n (%)	8 (4.9%)	14 (10.4%)	0.03*	0.36 (0.14-0.92)
Other, n (%)	4 (2.5%)	3 (2.2%)	0.84	0.86 (0.18-3.96)

No source, n (%)	116 (71.2%)	75 (56%)		
Polymicrobial BSI, n (%)	42 (25.8%)	28 (20.9%)	0.32	1.31 (0.76-2.27)
Candida sp. co-isolation, n (%)	11 (6.7%)	2 (15.4%)	0.043*	4.78 (1.04-21.9)
Acinetobacter sp. co-isolation, n (%)	14 (8.6%)	5 (3.7%)	0.1	2.42 (0.85-6.91)
Prior exposure				
Cephalosporins, n (%)	53 (32.5%)	55 (41%)	0.14	0.69 (0.43-1.11)
Clyndamicine, n (%)	8 (4.9%)	13 (9.7%)	0.07	0.44 (0.18-1.09)
Fluoroquinolones, n (%)	34 (20.9%)	25 (18.7%)	0.6	1.15 (0.64-2.04)
Linezolid, n (%)	37 (22.7%)	16 (11.9%)	0.02*	2.17 (1.14-4.1)
Piperacillin/tazobactam, n (%)	51 (31.7%)	23 (17.2%)	0.005*	2.24 (1.28-3.91)
Aminoglycosides, n (%)	5 (3.1%)	4 (3%)	0.98	1.01 (0.27-3.09)
Colistin, n (%)	42 (25.8%)	33 (24.8%)	0.85	1.05 (0.62-1.78)
Carbapenems, n (%)	73 (44.8%)	54 (40.3%)	0.43	1.2 (0.75-1.9)
Tigecycline, n (%)	7 (4.3%)	5 (3.7%)	0.8	1.15 (0.35-3.73)
Fluoroquinolones, n (%)	34 (20.9%)	25 (18.7%)	0.63	1.14 (0.64-2.04)
Metronidazole, n (%)	19 (11.7%)	19 (14.2%)	0.51	0.7 (0.4-1.57)
Penicillins, n (%)	10 (6.2%)	12 (9%)	0.37	0.67 (0.28-1.61)
Inappropriate initial therapy, n (%)	130 (79.8%)	97 (72.4%)	0.13	1.5 (0.87-2.57)
Initial therapy				
C/AVI (+/-AZT), n (%)	10 (6.1%)	20 (14.9%)	0.019*	0.37 (0.17-0.83)
Colistin, n (%)	62 (38%)	34 (25.4%)	0.02*	1.81 (1.09-2.98)
Others, n (%)	68 (41.7%)	59 (44%)	0.568	0.91 (0.57-1.44)
Fluoroquinolones, n (%)	12 (7.4%)	9 (6.7%)	0.825	1.12 (0.457-2.74)
Tigecycline, n (%)	12 (7.4%)	8 (6%)	0.65	1.27 (0.5-3.2)
Definitive therapy, n (%)				
C/AVI (+/-AZT), n (%)	40 (24.5%)	55 (41%)	0.009*	0.46 (0.28-0.76)
Colistin, n (%)	76 (46.6%)	51 (38.1%)		1.42 (0.9-2.26)
Others, n (%)	47 (28.8%)	28 (20.9%)		
		Laboratory results		
Urea (mg/dL), median (IQR)	77 [47.22-118.5]	53.45 [35.39-86.5]	<0.001	
Procalcitonine (ng/mL), median (IQR), N=292	7.46 [1.268-26.08]	1.645 [0.504-9.22]	<0.001	
Creatinine (mg/dL), median (IQR), N=295	1.2 [0.7-2.1]	0.74 [0.5-1.45]	<0.001	

C Reactive Protein (mg/L), median (IQR), N=282	180.25 [79.8-266.27]	129.35 [73.8-217.15]	0.03*
D-Dimers (ng/mL), median (IQR), N=198	1957 [896-3279]	957 [662-2482]	0.005*
Thrombocytes (10^3/uL), median (IQR)	172 [102.25-254.75]	215 [148.25-270]	0.003*
AST (U/L), median (IQR), N=282	50.5 [31-96.5]	37.5 [24.75-61.25]	<0.001*
ALT (U/L), median (IQR), N=282	30 [17-64.75]	30 [17.75-64.5]	0.798
Neutrophils (10^3/uL), median (IQR)	13.63 [8.11-19.29]	9.08 [6.77-14.13]	0.001*
Leucocytes (10^3/uL), median (IQR)	14.46 [9.28-20.2]	11.46 [8.37-16.3]	0.008*
Monocytes (10^3/uL), median (IQR)	0.42 [0.2-0.748]	0.6 [0.325-0.88]	0.013*
Erythrocytes (10^6/uL), median (IQR)	3.11 [2.63-3.57]	3.28 [2.81-3.85]	0.014*
Lymphocytes (10^3/uL), median (IQR)	0.64 [0.4-1.11]	0.91 [0.6-1.34]	<0.001*
Fibrinogen (mg/dL), median (IQR) N=278	426.5 [322-510.5]	460 [383.25-555.5]	0.047*
Hemoglobine (g/dL), median (IQR)	8.9 [7.62-10.4]	9.5 [8.4-11.25]	0.001*
Sodium (meq/mol), median (IQR)	140 [136-146]	139 [135-143]	0.058
Potassium (meq/mol), median (IQR)	3.9 [3.4-4.3]	3.8 [3.4-4.1]	0.167
Glucose (mg/dL), median (IQR)	111 [85-164]	111 [91-137]	0.832
Albumin (g/dL), median (IQR), N=210	2.6 [2.12-2.9]	2.6 [2.29-3]	0.169

*p<0.05

ALT: Alanine Aminotransferase; AST: Aspartate Aminotransferase; AZT: Aztreonam; BSI: bloodstream infection; CI95%: confidence interval 95%; ICU: Intensive Care Unit; IQR: Interquartilic range; C/AVI: ceftazidime/avibactam; CR: carbapenem-resistant; GI: gastroinetestinal; MBL: metallo beta lactamase; OR: odds ratio

Table S5. Multivariate analysis of factors associated to 14-day-mortality in bloodstream infections caused by Carbapenemase producing Enterobacterales

	B	S.E.	WALD	P	aOR (CI95%)
Age	0.001	0.012	0.002	0.96	1.00 (0.97-1.02)
Charlson	0.129	0.067	3.69	0.055	1.13 (0.99-1.29)
Vasopressor use	1.85	0.322	33.02	0.000*	6.37 (3.33-11.98)
ICU stay	0.51	0.359	2.055	0.152	1.67 (0.82-3.38)
Source of BSI (NO Source Ref.)			7.457	0.11	
Catheter	-1.17	0.555	4.45	0.035*	0.31 (0.1-0.92)
Respiratory	-0.97	0.493	3.865	0.049*	0.37 (0.29-0.99)
Urinary	-0.418	0.44	0.904	0.342	0.65 (0.27-1.55)
Other	-0.502	0.862	0.339	0.5	0.6 (0.11-3.27)
*Candidasp.*co-isolation	1.57	0.909	2.98	0.08	4.81 (0.81-28.55)
Inadequate initial therapy	0.535	0.365	2.155	0.14	1.7 (0.83-3.49)
Type of carbapenemase (single MBL Ref.)		1.66	4.36	0.64	
KPC	-0.424	0.45	0.88	0.34	0.65 (0.27-1.58)
OXA48	-0.478	0.48	0.98	0.32	0.62 (0.24-1.59)
OXA48/KPC+NDM	-0.099	0.45	0.047	0.82	0.9 (0.37-2.2)
Prior linezolid	1.002	0.416	3.31	0.016*	2.72 (1.2-6.15)
Prior piperacillin/tazobactam	0.615	0.338	3.311	0.06	1.84 (0.95-3.58)
Definitive therapy (Colistin therapy REF)			11.19	0.004*	
C/AVI(+/-AZT)	-1.006	0.356	7.98	0.005*	0.36 (0.18-0.73)
Others	0.31	0.37	0.7	0.4	1.36 (0.65-2.84)
Constant	-1.87	0.907	4.267	0.03	0.15

*$p<0.05$

aOR: **adjusted Odds Ratio**; AZT: **Aztreonam**; BSI: **bloodstream infection;** C/AVI: **ceftazidime/avibactam;**CI95%: **confidence interval 95%;** MBL: **metallo beta lactamase**; REF: **Reference**

Supplementary Tables S6–S10

Ceftazidime–Avibactam versus Colistin-Based Regimens for Carbapenem-Resistant Enterobacterales Bloodstream Infections: A Six-Year Retrospective Cohort and 30-Day Mortality Analysis

Victor-Pierre Ormeneanu, Anca Zanfirescu, Corina Andrei, Florin Dumitru, Flavius Anghel, Răzvan Adam and Simona Negreș

Table S6. Carbapenemase class, definitive therapy and 30-day mortality by calendar epoch (N=308)

Variable	2020–2021 (n=110)	2022–2023 (n=93)	2024–2025 (n=105)
KPC (incl. KPC+OXA-48), n (%)	62 (56.4%)	32 (34.4%)	7 (6.7%)
OXA-48-like, n (%)	31 (28.2%)	15 (16.1%)	25 (23.8%)
OXA-48/KPC+NDM, n (%)	5 (4.5%)	26 (28.0%)	59 (56.2%)

Variable	2020–2021 (n=110)	2022–2023 (n=93)	2024–2025 (n=105)
MBL alone, n (%)	12 (10.9%)	20 (21.5%)	14 (13.3%)
**MBL determinant present, %**	15.5%	49.5%	69.5%
Definitive C/AVI±AZT, n (%)	15 (13.6%)	21 (22.6%)	62 (59.0%)
Definitive colistin, n (%)	61 (55.5%)	50 (53.8%)	18 (17.1%)
Definitive other therapy, n (%)	34 (30.9%)	22 (23.7%)	25 (23.8%)
**30-day mortality (documented status), n/N (%)**	74/95 (77.9%)	59/84 (70.2%)	65/96 (67.7%)

Crude 30-day mortality by definitive regimen and epoch, patients with documented day-30 status:

Definitive regimen	2020–2021	2022–2023	2024–2025
C/AVI±AZT	10/12 (83.3%)	13/18 (72.2%)	34/56 (60.7%)
Colistin	43/53 (81.1%)	36/47 (76.6%)	13/16 (81.2%)

One record with an implausible year of inclusion (2033) was corrected to 2023 before analysis.

Table S7. Phenotypic ceftazidime/avibactam susceptibility, composition and timing of the definitive regimen, and polymicrobial bloodstream infection

Panel A. Phenotypic susceptibility by carbapenemase class (all 308 patients)

Carbapenemase class	Susceptible, n	Resistant, n	Not tested, n	% susceptible (of tested)
KPC (incl. KPC+OXA-48)	100	1	0	99.0%
OXA-48-like	71	0	0	100.0%
OXA-48/KPC+NDM	0	89	1	0.0%
MBL alone	1	38	7	2.6%
**No MBL determinant (KPC / OXA-48-like)**	**171**	**1**	**0**	**99.4%**
**MBL determinant present**	**1**	**127**	**8**	**0.8%**

The two discordant isolates and all eight untested isolates belonged to patients who received colistin or other therapies. Among the 202 patients entering the weighted comparison there was no discordance between enzyme class and phenotype.

Panel B. Composition of the definitive ceftazidime/avibactam-based regimen (n=98)

Regimen	n (%)	MBL determinant, n	Day-30 status documented, n	30-day deaths, n (%)
C/AVI monotherapy	29 (29.6%)	5	23	14 (60.9%)
C/AVI + colistin	35 (35.7%)	11	33	26 (78.8%)

Regimen	n (%)	MBL determinant, n	Day-30 status documented, n	30-day deaths, n (%)
C/AVI + aztreonam	15 (15.3%)	15	12	5 (41.7%)
C/AVI + aztreonam + colistin	19 (19.4%)	19	18	12 (66.7%)

Aztreonam was given only to patients whose isolate carried a metallo-β-lactamase determinant (34/34, 100%). Median duration of definitive therapy 10 days (IQR 6–13).

Panel C. Definitive regimen and 30-day mortality, colistin-based therapy as reference (n=202, 149 deaths)

Regimen	n	Deaths, n (%)	aOR (95% CI)	p	Firth aOR (95% CI)
Colistin-based (reference)	116	92 (79.3%)	1	—	1
C/AVI monotherapy	23	14 (60.9%)	0.27 (0.09–0.82)	0.021	0.29 (0.10–0.85)
C/AVI + colistin	33	26 (78.8%)	0.96 (0.33–2.81)	0.942	0.93 (0.34–2.74)
C/AVI + aztreonam	12	5 (41.7%)	0.09 (0.02–0.43)	0.003	0.11 (0.02–0.46)
C/AVI + aztreonam + colistin	18	12 (66.7%)	0.50 (0.12–2.05)	0.337	0.52 (0.14–2.04)

Adjusted for age, Charlson Comorbidity Index, carbapenemase class, vasopressor requirement, intensive-care stay and source of bloodstream infection. Firth column gives penalised-likelihood estimates with profile confidence intervals. The aztreonam-only group contains 12 patients with a documented day-30 status; its estimate is imprecise and exploratory.

Panel D. Interval from index blood culture to start of ceftazidime/avibactam (n=83 with both documented)

Interval	n	Deaths, n (%)
0–1 day	36	22 (61.1%)
2 days	13	9 (69.2%)
>2 days	34	25 (73.5%)

Median interval 2 days (IQR 0–3); χ^2 p=0.54. Adjusted odds ratio per additional day 1.12 (95% CI 0.85–1.46; p=0.42), adjusted for age, Charlson Comorbidity Index, carbapenemase class, vasopressor requirement and intensive-care stay.

Panel E. Polymicrobial bloodstream infection (N=308)

Co-isolated organism	n	% of cohort	% of polymicrobial episodes
Other Enterobacterales	26	8.4%	36.6%
Acinetobacter baumannii	20	6.5%	28.2%
Candida spp.	13	4.2%	18.3%
Gram-positive cocci	8	2.6%	11.3%
Pseudomonas aeruginosa	7	2.3%	9.9%

Co-isolated organism	n	% of cohort	% of polymicrobial episodes
**Any polymicrobial infection**	**71**	**23.1%**	—

Analysis	n	Result
30-day mortality, polymicrobial	65	48 (73.8%)
30-day mortality, monomicrobial	210	150 (71.4%)
Comparison	275	p=0.75
Distribution across definitive regimens	308	C/AVI±AZT 27, colistin 31, other 13; p=0.18

Weighted analysis	n	OR	95% CI	p
Polymicrobial status added to propensity score	202	0.51	0.27–0.97	0.040
Monomicrobial stratum	149	0.53	0.25–1.13	0.098
Polymicrobial stratum	53	0.45	0.14–1.47	0.184

Crude 30-day mortality by stratum and arm: monomicrobial, 39/61 (63.9%) under C/AVI±AZT versus 71/88 (80.7%) under colistin; polymicrobial, 18/25 (72.0%) versus 21/28 (75.0%). The polymicrobial stratum contains 53 patients and its estimate is imprecise.

Timing of aztreonam	Median (IQR), days
Index culture to start of ceftazidime/avibactam, with aztreonam	2 (1–3)
Index culture to start of ceftazidime/avibactam, without aztreonam	2 (0–4)

Mann–Whitney p=0.99. Carbapenemase class was established by rapid immunoassay and multiplex PCR on the positive blood culture, so aztreonam was started together with ceftazidime/avibactam once the determinant was identified, never as a later escalation, and only in patients whose isolate carried a metallo-β-lactamase determinant (34/34 in the ceftazidime/avibactam arm). Synergy testing was not performed.

Table S8. Weighted analysis of 30-day mortality, C/AVI±AZT versus colistin, adjusted for and stratified by calendar period

Analysis	n	OR	95% CI	p
Primary weighted analysis (Table 6)	202	0.49	0.26–0.93	0.028
Calendar period added to propensity score and outcome model	202	0.72	0.37–1.42	0.347
Stratum 2020–2021	65	1.14	0.17–7.69	0.890
Stratum 2022–2023	65	0.75	0.20–2.73	0.669
Stratum 2024–2025	72	0.44	0.16–1.21	0.113

The 2020–2021 stratum includes only 12 patients treated with C/AVI±AZT and the corresponding estimate is unstable.

Table S9. Weighted analysis of 30-day mortality stratified by carbapenemase class

Stratum	n	C/AVI±AZT	Colistin	OR	95% CI	p
OXA-48-like and/or KPC, no MBL determinant	110	27/40 (67.5%)	56/70 (80.0%)	0.48	0.20–1.15	0.100

Stratum	n	C/AVI±AZT	Colistin	OR	95% CI	p
MBL determinant present	92	30/46 (65.2%)	36/46 (78.3%)	0.46	0.18–1.20	0.113

Every isolate in the OXA-48-like/KPC stratum was phenotypically confirmed susceptible to ceftazidime/avibactam (Table S7, Panel A), so this stratum is simultaneously a phenotype-confirmed sensitivity analysis.

Table S10. Comparison of patients with and without a documented day-30 status, and extreme-case sensitivity analyses

Variable	Documented status (n=275)	Undocumented status (n=33)	p
Age, median (IQR)	69 (58-76)	71 (57-77)	0.97
Charlson Comorbidity Index, median (IQR)	6 (4-8)	7 (4-8)	0.58
Vasopressor requirement, n (%)	126 (45.8%)	5 (15.2%)	<0.001
Intensive-care stay, n (%)	207 (75.3%)	12 (36.4%)	<0.001
MBL determinant, n (%)	126 (45.8%)	10 (30.3%)	0.098
Unidentified source, n (%)	184 (66.9%)	12 (36.4%)	<0.001
Central-line source, n (%)	18 (6.5%)	6 (18.2%)	0.031
Urinary source, n (%)	39 (14.2%)	10 (30.3%)	0.024
Respiratory source, n (%)	29 (10.5%)	4 (12.1%)	0.766
Viral pneumonia, n (%)	65 (23.6%)	6 (18.2%)	0.662
Inappropriate initial therapy, n (%)	211 (76.7%)	23 (69.7%)	0.391
Definitive therapy: C/AVI, n (%)	86 (31.3%)	12 (36.4%)	0.84
Definitive therapy: colistin, n (%)	116 (42.2%)	13 (39.4%)	
Definitive therapy: other, n (%)	73 (26.5%)	8 (24.2%)	
Calendar period 2020-2021, n (%)	95 (34.5%)	15 (45.5%)	0.45
Calendar period 2022-2023, n (%)	84 (30.5%)	9 (27.3%)	
Calendar period 2024-2025, n (%)	96 (34.9%)	9 (27.3%)	

Extreme-case weighted analysis	n	OR	95% CI	p
All patients of unknown status assumed to have survived	227	0.56	0.32–0.98	0.044
All patients of unknown status assumed to have died	227	0.56	0.30–1.04	0.068

Weighted analyses use stabilised inverse-probability-of-treatment weights from a propensity score containing age, Charlson Comorbidity Index, presence of a metallo-β-lactamase determinant, vasopressor requirement, intensive-care stay and source of bloodstream infection. Re-estimation of the primary analysis within this framework gives OR 0.50 (95% CI 0.26–0.95) against OR 0.49 (95% CI 0.26–0.93) in Table 6; the difference does not affect interpretation.
